# The L-Arginine Transporter Solute Carrier Family 7 Member 2 Mediates the Immunopathogenesis of Attaching and Effacing Bacteria

**DOI:** 10.1371/journal.ppat.1005984

**Published:** 2016-10-26

**Authors:** Kshipra Singh, Nicole T. Al-Greene, Thomas G. Verriere, Lori A. Coburn, Mohammad Asim, Daniel P. Barry, Margaret M. Allaman, Dana M. Hardbower, Alberto G. Delgado, M. Blanca Piazuelo, Bruce A. Vallance, Alain P. Gobert, Keith T. Wilson

**Affiliations:** 1 Division of Gastroenterology, Hepatology, and Nutrition, Department of Medicine, Vanderbilt University Medical Center, Nashville, Tennessee, United States of America; 2 Center for Mucosal Inflammation and Cancer, Vanderbilt University Medical Center, Nashville, Tennessee, United States of America; 3 Veterans Affairs Tennessee Valley Healthcare System, Nashville, Tennessee, United States of America; 4 Department of Pathology, Microbiology, and Immunology, Vanderbilt University Medical Center, Nashville, Tennessee, United States of America; 5 Division of Gastroenterology, Department of Pediatrics, Child and Family Research Institute, University of British Columbia, Vancouver, British Columbia, Canada; 6 Department of Cancer Biology, Vanderbilt University Medical Center, Nashville, Tennessee, United States of America; University of California Davis School of Medicine, UNITED STATES

## Abstract

Solute carrier family 7 member 2 (SLC7A2) is an inducible transporter of the semi-essential amino acid L-arginine (L-Arg), which has been implicated in immune responses to pathogens. We assessed the role of SLC7A2 in murine infection with *Citrobacter rodentium*, an attaching and effacing enteric pathogen that causes colitis. Induction of SLC7A2 was upregulated in colitis tissues, and localized predominantly to colonic epithelial cells. Compared to wild-type mice, *Slc7a2* ^–/–^mice infected with *C*. *rodentium* had improved survival and decreased weight loss, colon weight, and histologic injury; this was associated with decreased colonic macrophages, dendritic cells, granulocytes, and Th1 and Th17 cells. In infected *Slc7a2* ^–/–^mice, there were decreased levels of the proinflammatory cytokines G-CSF, TNF-α, IL-1α, IL-1β, and the chemokines CXCL1, CCL2, CCL3, CCL4, CXCL2, and CCL5. In bone marrow chimeras, the recipient genotype drove the colitis phenotype, indicative of the importance of epithelial, rather than myeloid SLC7A2. Mice lacking *Slc7a2* exhibited reduced adherence of *C*. *rodentium* to the colonic epithelium and decreased expression of Talin-1, a focal adhesion protein involved in the attachment of the bacterium. The importance of SLC7A2 and Talin-1 in the intimate attachment of *C*. *rodentium* and induction of inflammatory response was confirmed *in vitro*, using conditionally-immortalized young adult mouse colon (YAMC) cells with shRNA knockdown of *Slc7a2* or *Tln1*. Inhibition of L-Arg uptake with the competitive inhibitor, L-lysine (L-Lys), also prevented attachment of *C*. *rodentium* and chemokine expression. L-Lys and siRNA knockdown confirmed the role of L-Arg and SLC7A2 in human Caco-2 cells co-cultured with enteropathogenic *Escherichia coli*. Overexpression of *SLC7A2* in human embryonic kidney cells increased bacterial adherence and chemokine expression. Taken together, our data indicate that *C*. *rodentium* enhances its own pathogenicity by inducing the expression of SLC7A2 to favor its attachment to the epithelium and thus create its ecological niche.

## Author Summary

Intestinal infections by attaching and effacing (A/E) bacteria widely impact human health, with major social and economic repercussions. Mucosal immunity plays a critical role in determining the outcome of these infections. The amino acid L-arginine regulates inflammatory responses to bacterial pathogens. We studied the role of the L-arginine transporter solute carrier family 7 member 2 (SLC7A2) during infection with the A/E pathogen *Citrobacter rodentium*. SLC7A2 is induced in colonic epithelial cells during the infection and facilitates the intimate attachment of the bacteria, thus initiating the inflammatory response of the infected mucosa. These data were confirmed *in vitro* using *C*. *rodentium*-infected mouse cells and human colonic epithelial cells infected with enteropathogenic *Escherichia coli*. Our work describes a mechanism by which A/E bacteria manipulate host response to favor their colonization, thereby positioning SLC7A2 as an unrecognized therapeutic target to limit infection with enterobacteria.

## Introduction

L-arginine (L-Arg) is a semi-essential amino acid whose metabolism can be dysregulated under diseased conditions. Transport of L-Arg is primarily dependent on the *y* ^+^ transport system, which includes the cationic amino acid transporter (CAT; SLC7A) family of proteins [1]. SLC7A1 is constitutively expressed and involved in basic metabolism [2, 3]; SLC7A2 is the inducible isoform, and includes the alternatively spliced isoforms SLC7A2A, a low-affinity transporter primarily in liver, and SLC7A2B, the high-affinity L-Arg transporter known to be abundant in macrophages [4–6]. Thus, SLC7A2-dependent cellular bioavailability of L-Arg controls the activity of two major enzymes of the innate response under pathophysiological processes: first, L-Arg is converted into nitric oxide (NO) by the inducible NO synthase (NOS2); second, arginase 1 and arginase 2 catabolize L-Arg into urea and L-ornithine, which serves as substrate for ornithine decarboxylase (ODC) and ornithine aminotransferase for the synthesis of polyamines and proline, respectively. In this context, our work has highlighted that SLC7A2 is a critical regulator of the inflammatory processes of the gastrointestinal tract [6–8]. We reported that generation of antimicrobial NO by macrophages exposed to the gastric pathogen *Helicobacter pylori* required SLC7A2 [6], and that mice deficient in S*lc7a2* exhibit attenuation of innate and adaptive immune responses to chronic infection with *H*. *pylori*, leading to less gastric inflammation [9]. In contrast, expression of macrophage SLC7A2 in the colonic tissue protected mice from dextran sulfate sodium (DSS)-induced colitis [10]. However, the expression and the role of SLC7A2 in colonic inflammation following infection with intestinal pathogenic bacteria has not been identified.

Bacterial pathogenesis is orchestrated by the targeted deleterious effects of virulence factors and by the molecular crosstalk between the host and the pathogen. In this aspect, non-invasive attaching-effacing (A/E) enteropathogens represent exquisite examples of bacteria that have acquired the ability to highjack host metabolism and responses. The A/E phenotype shared by enteropathogenic *Escherichia coli* (EPEC), enterohemorrhagic *E*. *coli*, and by a natural mouse pathogen, *Citrobacter rodentium*, is characterized by intimate adherence of bacteria to intestinal epithelial cells and by effacement of microvilli. Most of the proteins involved in the A/E phenotype are encoded on a pathogenicity island called the locus of enterocyte effacement (LEE) encoding a type III secretion system (T3SS) that allows bacteria to directly inject effector proteins into the underlying colonic epithelial cells (CECs) [11]. Thus, once in CECs, the bacterial translocated intimin receptor Tir is integrated into the plasma membrane and forms a hairpin-loop; the extracellular domain of Tir then binds intimin, an adhesin present at the bacterial cell surface [12]. The binding of intimin induces clustering of Tir, resulting in phosphorylation of Tir at tyrosine 471 [13, 14] and subsequent recruitment of proteins involved in actin polymerization and pedestal formation [15]. Of note, the phosphorylation of Tir is required for pedestal formation *in vitro*, but is not essential for colonization of mice by *C*. *rodentium* [14]. It has been also described that the intracellular N-terminus and C-terminus domains of EPEC Tir are also connected to focal adhesion proteins including vinculin, α-actinin, and talin [16]. These proteins, which are recruited independently of Tir phosphorylation [17], are required for pedestal formation [18] and thus have been immunolocalized beneath the attached bacteria [16]. Hence, focal adhesion proteins are essential for the attachment of EPEC to the host cell surface.

Moreover, the bacterial effectors injected into cells through the T3SS, components of the translocation machinery, and/or T3SS-secreted non-LEE-encoded effectors, interfere with the host signal transduction and may thus modulate tight junction disruption [19] and innate immune response [20–22]. Therefore, the capacity of A/E bacteria to attach intimately CECs is a critical step in pathogenesis.

Here we determined that *C*. *rodentium* induces *Slc7a2* mRNA and SLC7A2 protein expression in CECs of infected mice. Genetic ablation of *Slc7a2* caused a significant reduction of colonization of the colonic mucosa, associated with reduced expression of Talin-1, resulting in improved clinical, histopathological, and immunological parameters. Finally, we also demonstrated that SLC7A2 favors the intimate attachment of *C*. *rodentium* and EPEC to epithelial cells and, consequently, the induction of the innate immune response.

## Results

### SLC7A2 expression is upregulated in colonocytes during *C*. *rodentium* infection

**Fig 1:**
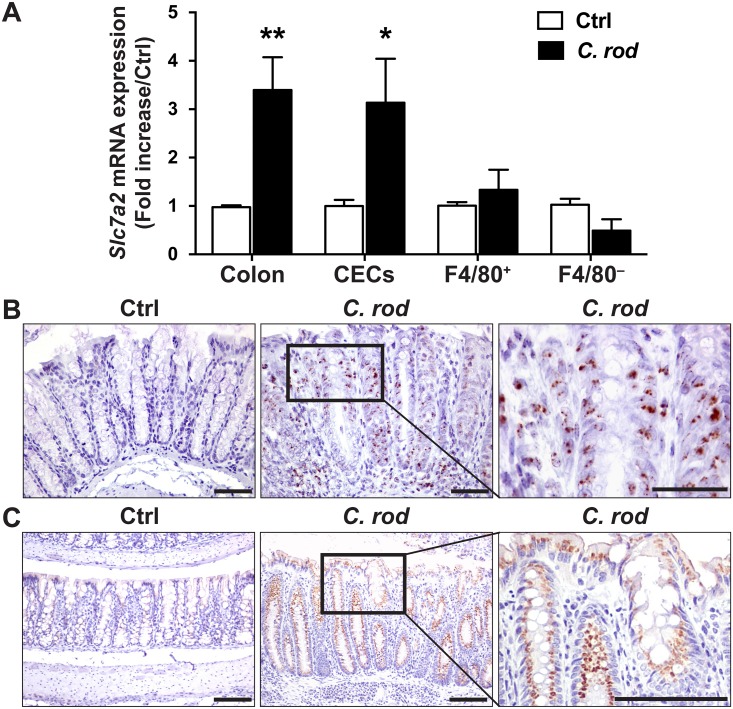
Induction of *Slc7a2* mRNA and SLC7A2 protein expression by *C*. *rodentium* in colon tissues. C57BL/6 mice were infected with *C*. *rodentium* (*C*. *rod*) or not (Ctrl) and colonic tissues were harvested after 14 days. (**A**) Levels of *Slc7a2* mRNA in whole colon tissue, CECs, and F4/80^+^ and F4/80^−^ lamina propria cells. *P < 0.05, **P < 0.01 vs. Ctrl; *n* = 3–7 mice for Ctrl, and *n* = 5–11 for infected animals. (**B**) RNAscope for *Slc7a2* in colon tissues, Scale bar = 50 μm. Representative staining of *n* = 2 for Ctrl and *n* = 6 for *C*. *rodentium*-infected mice. (**C**) Colon sections were stained for SLC7A2 protein by immunohistochemistry. Scale bar = 100 μm. Representative image of *n* = 3 for Ctrl and *n* = 5 for *C*. *rodentium*-infected mice.

When compared to uninfected mice, there was a significant increase in *Slc7a2* mRNA levels in colonic tissues from *C*. *rodentium*-infected mice (Fig 1A). To further determine the cellular localization of *C*. *rodentium*-induced *Slc7a2* mRNA, we analyzed the expression of this gene in various cells purified from the colon. *Slc7a2* mRNA levels were increased in isolated CECs to the same extent as in whole tissues (Fig 1A); in contrast, this gene was not induced in F4/80^+^ (macrophages) or F4/80^−^ lamina propria cells (Fig 1A). We also examined mRNA levels of *Slc7a2* by *in situ* hybridization (RNAscope), and found increased *Slc7a2* mRNA levels in infected mice, predominantly in CECs (Fig 1B), but some cells from the lamina propria were also stained (Fig 1B). Moreover, *Slc7a2* mRNA levels were not detectable in infected *Slc7a2* ^−/−^mice (S1A Fig). Importantly, *Slc7a1* mRNA expression was not increased in colonic tissues from infected WT and *Slc7a2* ^–/–^mice when compared to the respective uninfected control animals (S1B Fig).

Immunohistochemistry demonstrated that SLC7A2 protein was mainly increased in CECs from mice infected with *C*. *rodentium* compared to those that were uninfected (Fig 1C). This was confirmed by Western blot analysis showing increased SLC7A2 protein levels in colonic epithelial cells of wild-type mice infected with *C*. *rodentium* compared to uninfected animals (S1C Fig); SLC7A2 was not detected in uninfected and infected *Slc7a2* ^–/–^mice (S1C Fig). As expected, L-Arg uptake in colonic tissue was increased in *C*. *rodentium*-infected wild-type mice compared to uninfected animals (S1D Fig); this was completely suppressed in *Slc7a2* ^–/–^mice (S1D Fig). Consistent with the decreased tissue L-Arg uptake, L-Arg concentration was significantly higher in the serum of *Slc7a2* ^*–/–*^mice compared to wild-type mice, for both uninfected and infected mice (S1E Fig).

Lastly, we analyzed *Slc7a2* mRNA expression in the conditionally-immortalized young adult mouse colon (YAMC) cell line. The expression of *Slc7a2* was induced at the same level by *C*. *rodentium* or by mutant strains lacking *escN*, *espF*, or *espG* (S2A Fig), suggesting that the T3SS and the injected effector proteins are not involved in *Slc7a2* induction. However, the *escN* mutant failed to stimulate *Cxcl1* and *Cxcl2* expression, whereas the *espF* and *espG* mutants induced the expression of these genes at the same level as the wild type *C*. *rodentium* (S2B Fig).

### *Slc7a2* ^–/–^mice are protected from *C*. *rodentium* colitis

**Fig 2:**
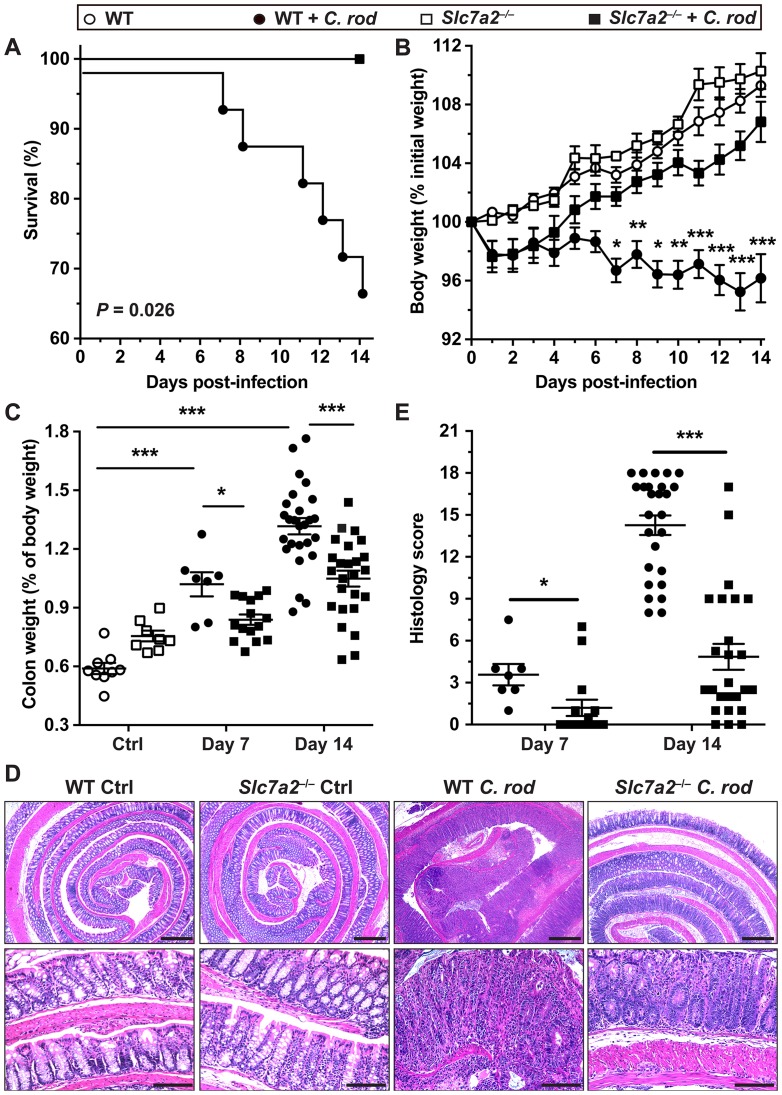
Effect of *Slc7a2* deletion on *C*. *rodentium* colitis. C57BL/6 or *Slc7a2* ^–/–^mice were infected with *C*. *rodentium* (*C*. *rod*). (**A**) Survival was monitored daily; the Kaplan–Meier plot was performed from 5 separate experiments (*n* = 33 WT and *n* = 30 *Slc7a2* ^–/–^mice); no death was observed in uninfected animals. (**B**) Body weights of all the animals in Panel (**A**) were measured daily and are presented as percentage of initial body weight. *P < 0.05, **P < 0.01, ***P < 0.001 compared to uninfected animals with the same genotype. (**C**) After euthanasia, colons were harvested, washed, and weighed. Values are expressed as percent of body weight on the day of sacrifice. (**D-E**) Colons were Swiss-rolled and stained with H&E (**D**) and scored for histologic injury (**E**); Scale Bar, 100 μm. For *C* and *E*, *P < 0.05, **P < 0.01, ***P < 0.001; the data depicted for Day 14 are from the same mice presented in (**A**) and the data at Day 7 is derived from a separate experiment.

Because *Slc7a2* was induced in colonocytes by *C*. *rodentium*, we sought to determine the effect of *Slc7a2* genetic deletion on the course of the infection. As shown in Fig 2A, *Slc7a2* ^–/–^mice survived throughout the experiment while death of WT mice began on day 7 and continued until day 14, indicating a significant protective effect of *Slc7a2* deletion. Mice of both genotypes began losing weight on day 1, but *Slc7a2* ^–/–^mice started gaining weight after day 3, indicating recovery, and had less weight loss on days 6–14 (Fig 2B). We also measured the weight of the colons, as thickening of the colon is an indicator of colitis severity in this model. Colon weight was increased in WT mice infected with *C*. *rodentium* compared to uninfected controls (Fig 2C), and this was significantly attenuated in infected *Slc7a2* ^–/–^mice compared to infected WT mice by 15.5 ± 3.8% and 20.1 ± 3.2% at day 7 and 14, respectively.

Photomicrographs of H&E staining of the colons of *C*. *rodentium*-infected WT mice demonstrate a complete effacement of the brush border microvilli, massive crypt hyperplasia, severe mucosal inflammation, and submucosal edema (Fig 2D); however, the epithelial injury and the inflammation were both markedly attenuated in infected *Slc7a2* ^–/–^mice (Fig 2D). Using a comprehensive scoring system to quantify the degree of inflammation and epithelial damage, there was a significant decrease in overall histologic injury in *Slc7a2* ^–/–^mice compared to WT animals at both day 7 and at day 14 (Fig 2E). Furthermore, loss of colonic goblet cells is another characteristic in mice infected with *C*. *rodentium* [23]; when assessed by using periodic acid–Schiff staining, we found that infection resulted in a 47.5 ± 6.5% loss of goblet cells in WT mice compared to only a 25.6 ± 5.3% loss in *Slc7a2* ^–/–^mice (S3 Fig). There was no detectable inflammation or epithelial injury in uninfected *Slc7a2* ^–/–^or WT mice (Fig 2D and S3 Fig).

### *C*. *rodentium* colitis is not mediated by SLC7A2 from hematopoietic cells

**Fig 3:**
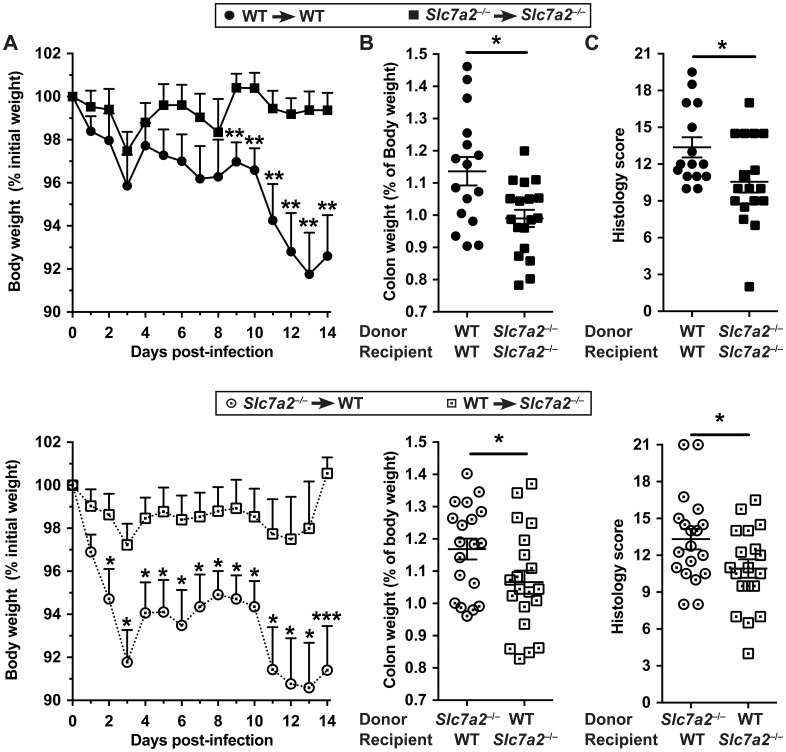
Bone marrow transplant between WT and *Slc7a2* ^–/–^mice. Irradiated animals were given bone marrow-derived hematopoietic cells and were infected with *C*. *rodentium*. Body weight (**A**), colon weight (**B**) and histologic scores (**C**) were determined. *P < 0.05, **P < 0.01, ***P < 0.001.

While SLC7A2 is known as a macrophage L-Arg transporter [4], our data indicate that upregulation of SLC7A2 during infectious colitis was mainly observed in the epithelium. Therefore, to further refine the contributions of different cells to the disease process, we infected WT and *Slc7a2* ^–/–^mice with *C*. *rodentium* 8 weeks following bone marrow transplants. We confirmed by genotyping the efficiency of the transplantation (S4 Fig). Similar to what we had observed with non-transplanted mice, *Slc7a2* ^–/–^receiving *Slc7a2* ^–/–^marrow had less body weight loss (Fig 3A), colon weight (Fig 3B), and histological damage (Fig 3C) than WT mice receiving WT marrow. Importantly, each of these clinical parameters were significantly improved in infected *Slc7a2* ^–/–^mice that received WT marrow, when compared to WT mice that received *Slc7a2* ^–/–^marrow (Fig 3). There was no detectable weight loss, inflammation or epithelial injury in any of the uninfected control mice in the bone marrow transplant experiments. Taken together, these data show that protection from colitis in the *C*. *rodentium* model by *Slc7a2* deletion is mediated by the recipient mouse genotype; this indicates that exacerbation of colitis due to SLC7A2 derives from the non-hematopoietic cells, consistent with the predominantly epithelial SLC7A2 localization that we have observed.

### Decreased mucosal immune activation in *Slc7a2* ^–/–^mice

**Fig 4:**
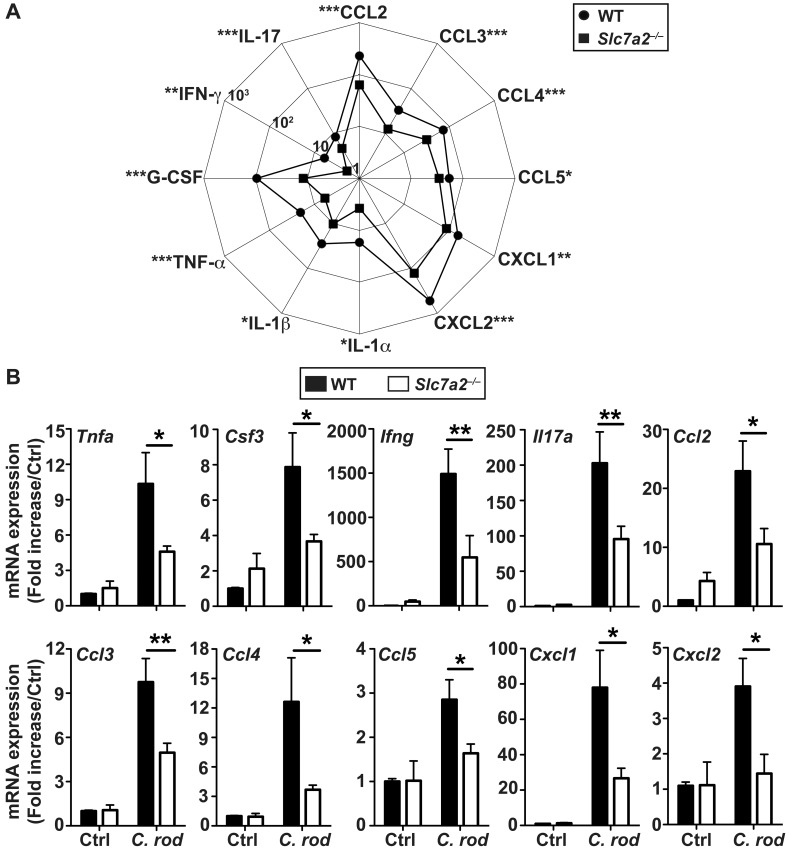
Immunoprofiling of WT and *Slc7a2* ^–/–^mice. Animals were infected with *C*. *rodentium* (*C*. *rod*) or not (Ctrl) for 14 days. (**A**) The concentrations of the cytokines in colon lysates were determined by Luminex assay. The scale represents the concentrations of analytes in pg/mg protein in colon tissues. *P < 0.05, **P < 0.01, ***P < 0.001 denote significant differences between infected WT (*n* = 14) and *Slc7a2* ^–/–^mice (*n* = 14). (**B**) Analysis of mRNA expression in the colon tissues. *P < 0.05, **P < 0.01; *n* = 3–5 Ctrl and *n* = 12–14 infected mice for each genotype.

To further investigate differences in inflammatory responses, we conducted cytokine profiling in colonic tissues by Luminex analysis. In WT mice, the chemokines CCL2, CCL3, CCL4, CCL5, CXCL1, and CXCL2, the innate pro-inflammatory cytokines TNF-α, IL-1α, IL-1β, and G-CSF, as well as the prototype Th1 and Th17 cytokines, IFN-γ and IL-17, respectively, were all significantly increased above the levels in infected mice in the colonic mucosa during *C*. *rodentium* infection (S1 Table). Notably, all of these immune effectors were produced in lower quantities by infected *Slc7a2* ^–/–^mice when compared to the WT animals (Fig 4A). These results were confirmed by analysis of the mRNA levels of the genes encoding theses chemokines and cytokines, showing a significant induction of gene expression in *C*. *rodentium*-infected WT mice vs. uninfected animals, and a significant reduction in these genes in *Slc7a2* ^–/–^animals (Fig 4B). In contrast, the Th2 cytokines IL-4, IL-5, and IL-13 and the immunoregulatory cytokine IL-10 showed no significant increase in *C*. *rodentium*-infected mucosa of WT or *Slc7a2*-deficient mice compared to the respective controls (S1 Table).

**Fig 5:**
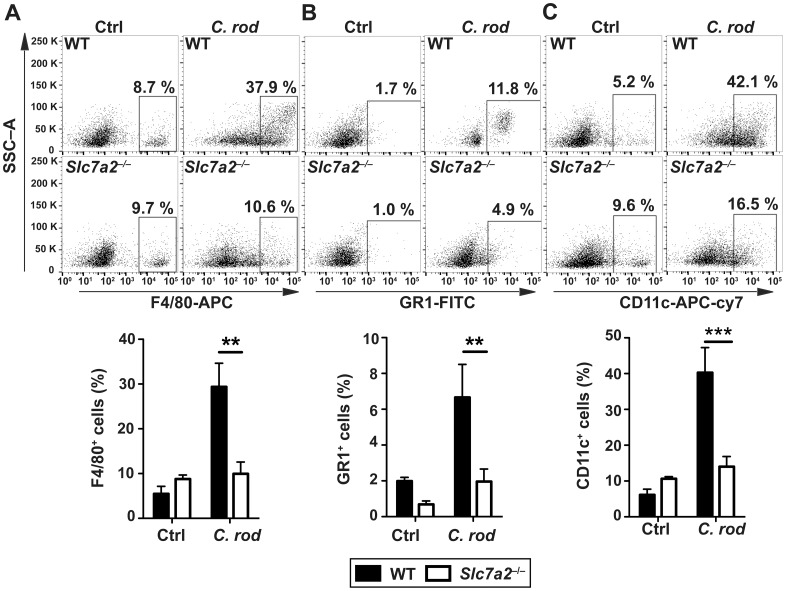
Immunophenotyping of innate immune cells. Cells isolated from the lamina propria of *C*. *rodentium*-infected mice (*C*. *rod*) or of uninfected animals were analyzed by flow cytometry for the following markers: (**A**) F4/80, (**B**) GR1, (**C**) CD11c. For each marker, representative flow plots and combined data from *n* = 4 Ctrl and *n* = 6 infected mice are shown **P < 0.01, ***P < 0.001.

**Fig 6:**
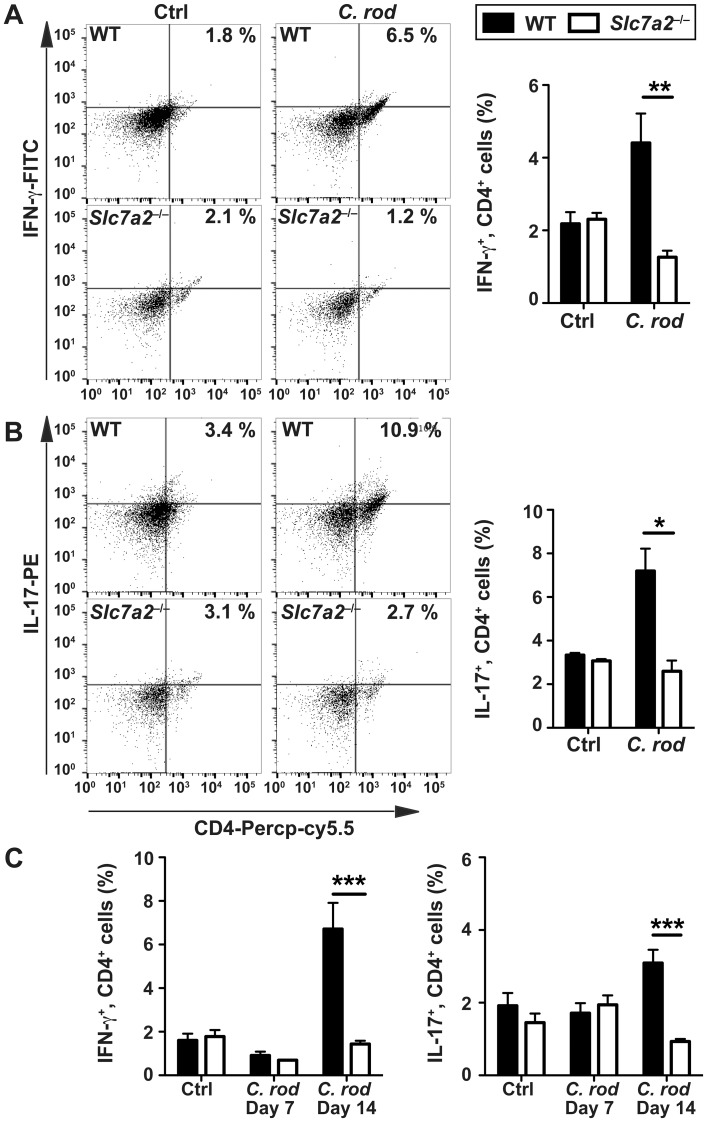
Immunophenotyping of T cells. Cells isolated from the lamina propria of *C*. *rodentium*-infected mice (*C*. *rod*) or of uninfected animals were analyzed by flow cytometry for CD4 and for IFN-γ (**A**) or IL-17 (**B**). For each marker, representative flow plots and combined data from *n* = 4 Ctrl and *n* = 6 infected mice are shown. (**C**) The same experiment was performed using mesenteric lymph node-derived cells. *P < 0.05, **P < 0.01, ***P < 0.001.

To establish the nature of the inflammatory cell populations in infected colonic tissues, we conducted immunophenotyping by flow cytometry. There were more F4/80^+^ cells (macrophages; Fig 5A), GR1^+^ cells (granulocytes; Fig 5B), and CD11c^+^ cells (dendritic cells; Fig 5C) in *C*. *rodentium*-infected WT animals compared to uninfected mice; each of these myeloid cells of the innate immune system were less abundant in the mucosa of infected *Slc7a2* ^−/−^mice (Fig 5A–5C). Similarly, CD4^+^, IFN-γ^+^ cells (Fig 6A) and CD4^+^, IL-17^+^ cells (Fig 6B) were more abundant in the colonic lamina propria and in the mesenteric lymph nodes (Fig 6C) of WT mice compared to *Slc7a2* ^−/−^mice during *C*. *rodentium* infection.

### *C*. *rodentium* colonization is reduced in mice lacking *Slc7a2*

**Fig 7:**
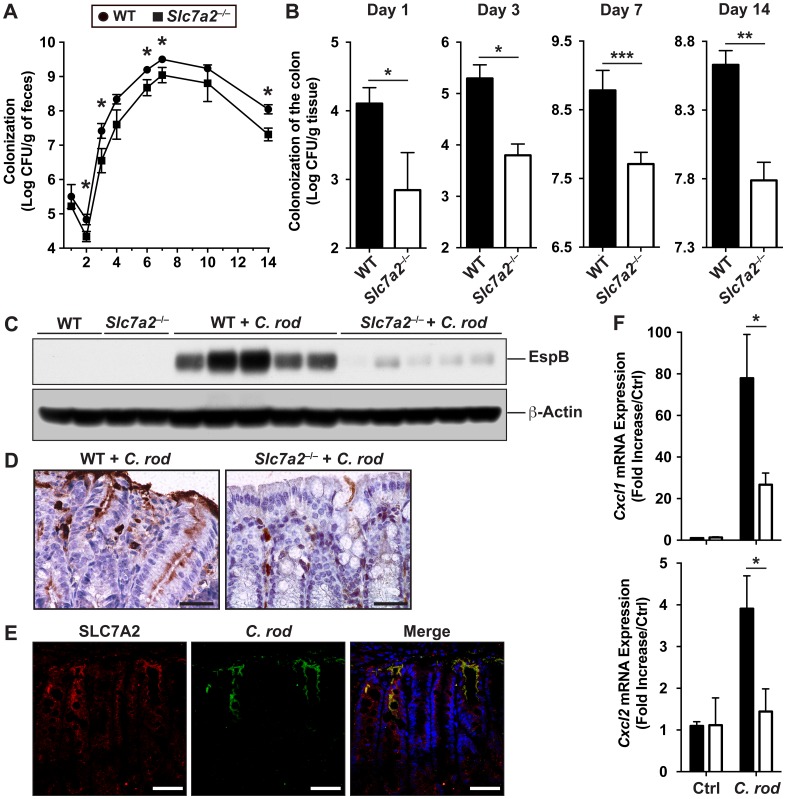
Colonization levels in WT and *Slc7a2* ^–/–^mice. (**A**) Fecal shedding of *C*. *rodentium* (*C*. *rod*). *P < 0.05 vs. infected *Slc7a2* ^–/–^mice; day 1, *n* = 9 WT and *n* = 9 *Slc7a2* ^–/–^; day 2, *n* = 15 WT and *n* = 9 *Slc7a2* ^–/–^; day 3, *n* = 17 WT and *n* = 11 *Slc7a2* ^–/–^; day 4, *n* = 11 WT and *n* = 6 *Slc7a2* ^–/–^; day 6–10, *n* = 11 WT and *n* = 8 *Slc7a2* ^–/–^; day 14, *n* = 20 WT and *n* = 21 *Slc7a2* ^–/–^. (**B**) Colonization of the colon by *C*. *rodentium*. *P < 0.05, **P < 0.01, ***P < 0.001; *n* = 9–28. (**C**) EspB and β-actin were detected in lysates of whole colonic tissues by Western blotting, each lane is from a different mouse infected (*C*. *rod*) or not (Ctrl) for 14 days. (**D**) Longitudinal sections of the colon were immunostained for EspB. Representative images from 5 animals in each group are shown; Scale bar, 100 μm. (**E**) The same sections were used for immunofluorescence for SLC7A2 (red), *C*. *rodentium* (green), and nuclei (blue); the merged image is shown (yellow). Scale bar = 20 μm. (**F**) *Cxcl1* and *Cxcl2* mRNA expression in isolated CECs from WT and *Slc7a2* ^–/–^mice infected or not with *C*. *rodentium*. *n* = 3 Ctrl and *n* = 5 infected mice.

During the time course of the infection, there was less *C*. *rodentium* excreted into the stool by *Slc7a2* ^–/–^mice in comparison to infected WT animals (Fig 7A). Importantly, the colonization of the colon by *C*. *rodentium* was decreased by more than a Log-order in *Slc7a2* ^–/–^mice compared to WT mice, at day 1, 3, 7 and 14 (Fig 7B). This was confirmed by detection of the bacterial protein EspB, a part of the T3SS [11], in the colonic tissues: By Western blot (Fig 7C) and immunohistochemistry (Fig 7D) there was markedly less EspB in *Slc7a2* ^–/–^versus WT tissues. Of importance, when WT animals were infected with *C*. *rodentium*, we found by immunofluorescence and confocal microscopy that the bacteria co-localized with SLC7A2 (Fig 7E).

We then reasoned that the decreased colonization level observed in *Slc7a2* ^–/–^mice could be linked to attenuation of the innate immune function of CECs. To verify this, we isolated CECs from mice and analyzed *Cxcl1* and *Cxcl2* mRNA expression. Both genes were induced in CECs of infected WT mice, but this was significantly reduced in *Slc7a2* ^–/–^mice compared to uninfected animals (Fig 7F).

### Talin-1 expression is downregulated in *Slc7a2* ^–/–^mice

**Fig 8:**
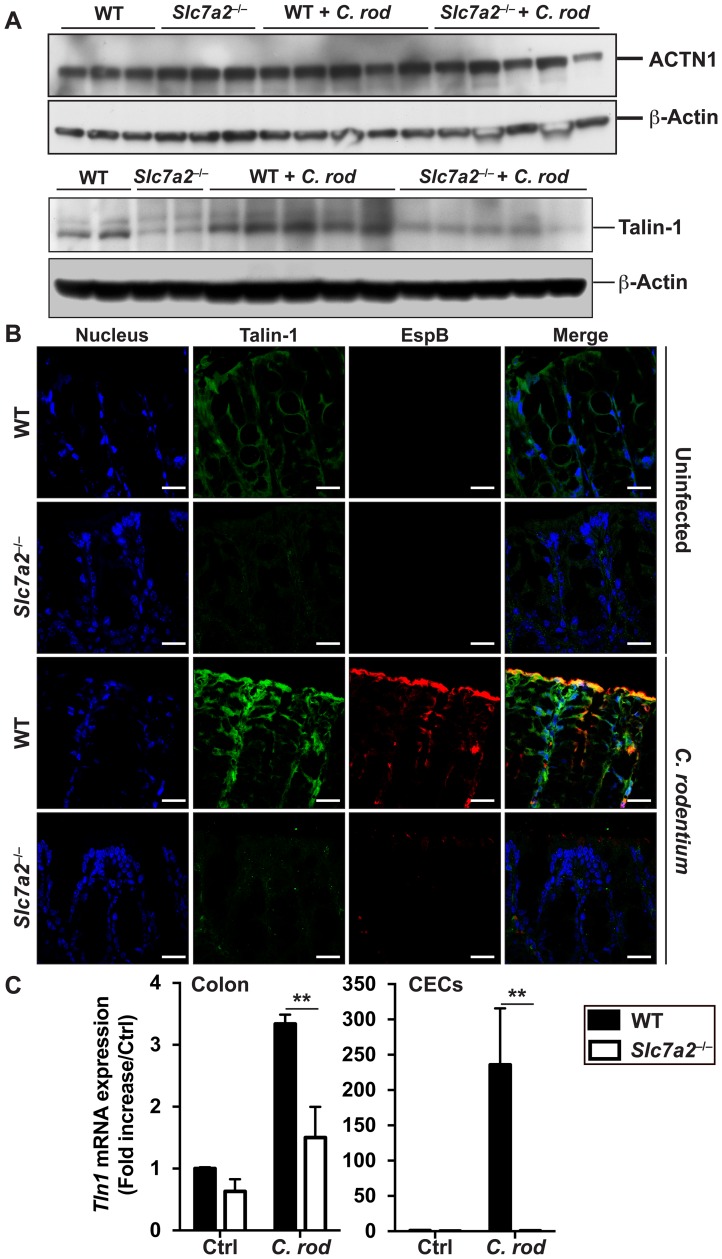
Analysis of focal contact proteins. WT or *Slc7a2* ^–/–^mice were infected with *C*. *rodentium* (*C*. *rod*) for 14 days. (**A**) ACTN1, Talin-1 and β-actin were detected in lysates of whole colonic tissues by Western blotting; each lane is from a different mouse. (**B**) Longitudinal sections of the colon were used for immunofluorescence for bacterial EspB (red), Talin-1 (green), and nuclei (blue); the merge of red and green is depicted in yellow. Representative images from 3 animals in each group are shown. Scale bar = 20 μm. (**C**) Expression of *Tln1* mRNA in whole colon tissue and CECs. **P < 0.01; *n* = 3 mice for Ctrl, and *n* = 5 for infected animals.

To understand the mechanism by which SLC7A2 may regulate colonization, we assessed the expression of host focal contact proteins, which are involved in the attachment of *C*. *rodentium* to epithelial cells independently of Tir phosphorylation [16, 17]. We observed that α-actinin (ACTN1) protein expression in the colon was not affected by *C*. *rodentium* infection nor by *Slc7a2* deletion (Fig 8A); in contrast, Talin-1 protein was less abundant in colonic tissues of *Slc7a2*-deficient mice, infected or not with *C*. *rodentium* (Fig 8A). This was confirmed by immunofluorescence (Fig 8B), showing an induction of Talin-1 protein expression in WT mice, but not in *Slc7a2* ^–/–^mice, infected with *C*. *rodentium* compared to uninfected controls. We then established that *Tln1* mRNA was induced in colonic tissues, and mainly in CECs, when WT mice were infected with *C*. *rodentium* (Fig 8C); in accordance to the proteins levels, we found that this gene was less upregulated in infected *Slc7a2* ^–/–^mice (Fig 8C).

### SLC7A2 triggers the attachment of *C*. *rodentium* to epithelial cells

**Fig 9:**
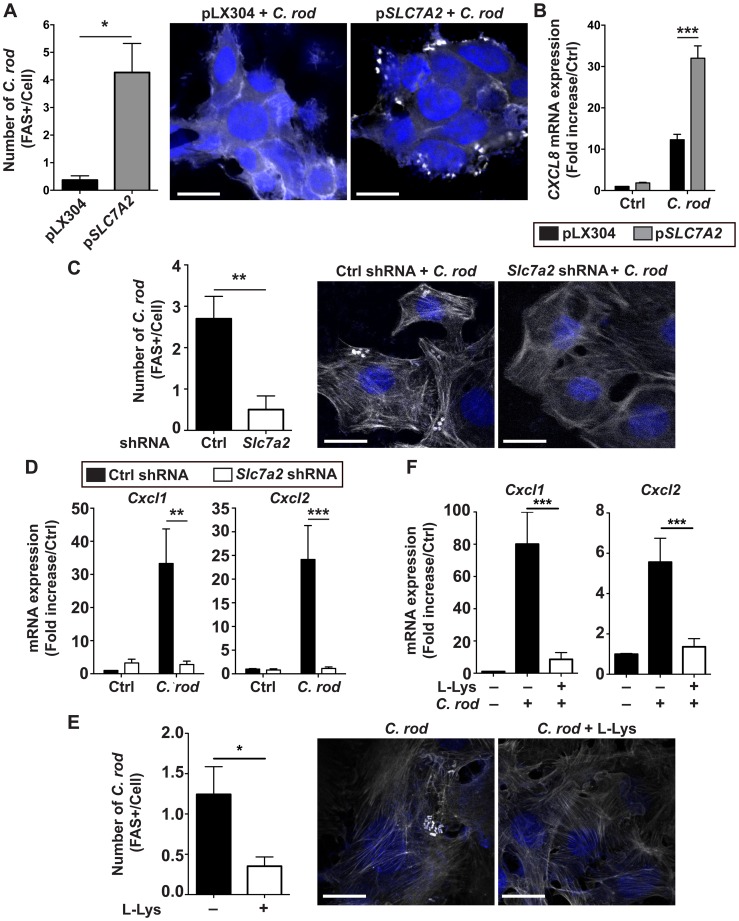
Effect of SLC7A2 on intimate attachment of *C*. *rodentium*. (**A**) Phalloidin staining in HEK 293T cells transfected with pLX304 or p*Slc7a2* and infected for 4 h with *C*. *rodentium*. *P < 0.05; representative images of 3 independent experiments are shown. Scale bar, 20 μm. (**B**) *CXCL8* mRNA in transfected HEK 293T cells in response to *C*. *rodentium*. ***P < 0.001; *n* = 3. (**C**) YAMC cells were transduced with Ctrl or *Slc7a2* shRNA, infected for 4 h with *C*. *rodentium* and stained for phalloidin. **P < 0.01; representative images of 3 independent experiments are shown. Scale bar, 10 μm. (**D**) *Cxcl1* and *Cxcl2* mRNA levels in YAMC cells with Ctrl or *Slc7a2* shRNA. **P < 0.01, ***P < 0.001; *n* = 3. (**E**) FAS test in YAMC cells infected for 4 h with *C*. *rodentium* in the presence or absence of L-Lys. *P < 0.05; representative images of 3 independent experiments are shown. Scale bar, 20 μm. (**F**) *Cxcl1* and *Cxcl2* mRNA expression in YAMC cells in response to *C*. *rodentium* ± L-Lys. ***P < 0.001; *n* = 3 independent experiments.

Because the *in vivo* data suggested that SLC7A2 favors colonic colonization by *C*. *rodentium*, we further investigated the role of this protein in the attachment of the bacteria to the cells. First, we used HEK 293T cells transfected with a pLX304 plasmid harboring the human *SLC7A2* gene; when compared to the cells transfected with empty vector, there was a concomitant increase in *SLC7A2* mRNA expression (S5A Fig) and formation of A/E lesions on the cells, assessed by fluorescence actin staining (FAS) test (Fig 9A). Moreover, the induction of the human *CXCL8* gene observed in infected HEK 293T cells was further increased in *SLC7A2*-overexpressing cells (Fig 9B).

Second, we used YAMC cells transduced with *Slc7a2* short-hairpin RNA (shRNA), and found more than 70% knockdown of *Slc7a2* mRNA expression compared to control shRNA-transduced uninfected cells (S5B Fig); the *Slc7a2* gene was induced by ~ 15 fold in *C*. *rodentium*–infected YAMC cells transduced with control shRNA, and the expression was essentially abolished in infected cells transduced with *Slc7a2* shRNA (S5B Fig). Similarly, *C*. *rodentium*-stimulated SLC7A2 protein expression was completely inhibited in YAMC cells expressing *Slc7a2* shRNA (S5C Fig). *Slc7a2* knockdown was accompanied by a marked inhibition of bacterial adhesion to cells (Fig 9C) and by a complete inhibition of *C*. *rodentium*-induced *Cxcl1* and *Cxcl2* mRNA expression (Fig 9D).

To determine whether these effects of SLC7A2 were mediated by L-Arg, we performed experiments with L-lysine (L-Lys), a competitor of L-Arg uptake by SLC7A2 [24]. As shown in Fig 9E, the attachment of *C*. *rodentium* to YAMC cells was decreased in the presence of L-Lys. In addition, the expression of the genes encoding CXCL1 and CXCL2 in *C*. *rodentium*-infected cells was inhibited by L-Lys (Fig 9F). Together, these data establish that SLC7A2 favors the attachment of *C*. *rodentium* by a mechanism involving L-Arg.

### L-Arg regulates talin-dependent adhesion of *C*. *rodentium* to CECs

**Fig 10:**
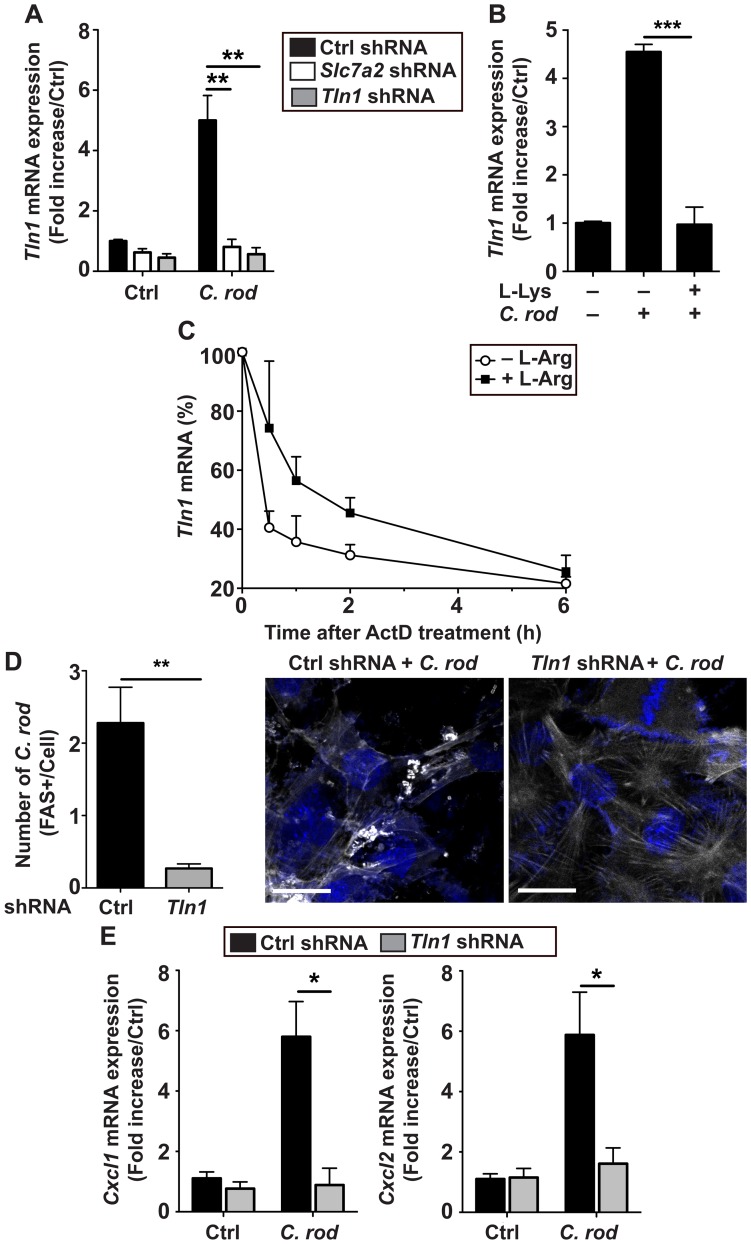
Effect of Talin-1 on intimate attachment of *C*. *rodentium*. (**A**) *Tln1* mRNA level in YAMC cells transduced with Ctrl, *Slc7a2*, or *Tln1* shRNA, infected or not with *C*. *rodentium* (*C*. *rod*). **P < 0.01; *n* = 3 independent experiments. (**B**) Effect of L-Lys on *Tln1* expression in YAMC cells. ***P < 0.001; *n* = 3 independent experiments. (**C**) Cells were L-Arg-depleted or not and treated with actinomycin D (ActD); total RNA was extracted at the indicated times and *Tln1* mRNA was analyzed by real-time PCR. The combined results of two independent experiments are shown and depicted as the percent of the *Tln1* expression compared to the level before addition of actinomycin D. (**D-E**) YAMC cells were transduced with Ctrl or *Tln1* shRNA infected with *C*. *rodentium*; FAS test (**D**) and *Cxcl1* and *Cxcl2* mRNA levels (**E**) were analyzed after 4 h. *P < 0.05, **P < 0.01; representative images of 3 independent experiments are shown. Scale bar, 20 μm.

Because Talin-1 protein is less abundant in *C*. *rodentium*-infected mice (Fig 8), we reasoned that endogenous L-Arg may affect Talin-1 expression and consequently bacterial adherence. First, we found that the gene *Tln1* that encodes Talin-1 was induced by 5–fold in YAMC cells expressing Ctrl shRNA and infected with *C*. *rodentium* compared to uninfected cells (Fig 10A). This induction of *Tln1* was abolished in cells transfected with *Slc7a2* shRNA or *Tln1* shRNA (Fig 10A). Further, *C*. *rodentium*-induced *Tln1* mRNA expression was also abolished in cells treated with L-Lys (Fig 10B). As depicted in Fig 10C, *Tln1* mRNA was more stable in YAMC cells in the presence of L-Arg (t_1/2_ = 80.32 min) than in cells starved of L-Arg (t_1/2_ = 19.02 min; *P* = 0.019).

Then, we observed a diminution of the attachment of *C*. *rodentium* to YAMC cells expressing *Tln1* shRNA compared to cells transfected with Ctrl shRNA (Fig 10D), demonstrating that Talin-1 is involved in the intimate binding of the bacteria to epithelial cells. A concomitant reduction of *C*. *rodentium*-induced *Cxcl1* and *Cxcl2* mRNA expression was also evidenced in cells with *Tln1* knockdown (Fig 10E).

### Adhesion of EPEC to human CECs is regulated by SLC7A2

**Fig 11:**
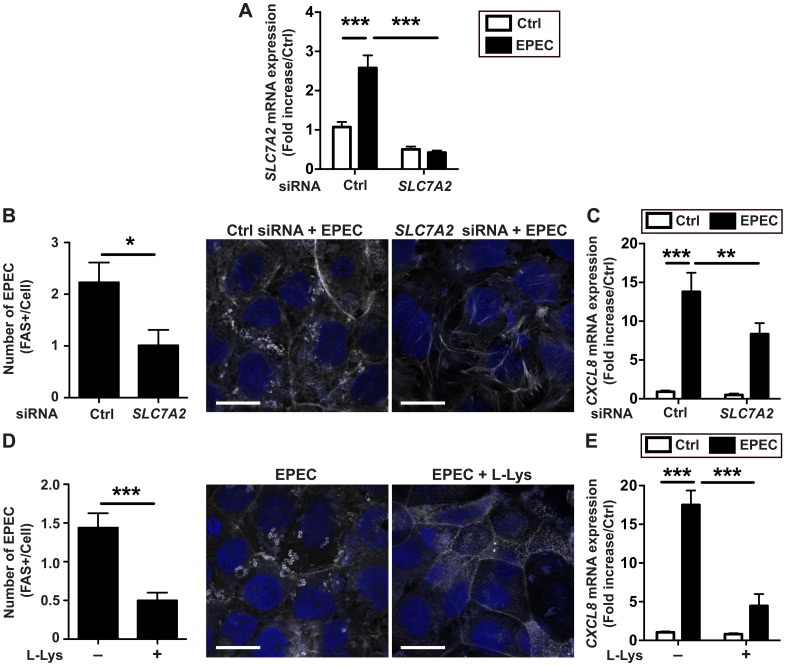
Expression and role of SLC7A2 in human CECs. *SLC7A2* mRNA levels (**A**), FAS test (**B**), and *CXCL8* mRNA levels (**C**) in Caco-2 cells transfected with control (Ctrl) or *SLC7A2* siRNA and infected for 4 h with EPEC. *P < 0.05, **P < 0.01, ***P < 0.001; *n* = 3 independent experiments. Scale bar, 20 μm. (**D-E**) Caco-2 cells were infected for 4 h with EPEC ± L- Lys and stained for phalloidin (**D**) or assessed *CXCL8* expression (**E**). ***P < 0.001; *n* = 3 independent experiments. In (B) and (D): scale bar, 20 μm.

We then recapitulated our findings in the human colonic epithelial cell line Caco-2 infected with the human pathogen EPEC. We observed that the gene *SLC7A2* was induced during infection with EPEC (Fig 11A); this expression was completely suppressed in cells transiently transfected with small interfering RNA (siRNA) directed against *SLC7A2* (Fig 11A). The adhesion of EPEC to Caco-2 cells (Fig 11B and 11D) and the EPEC-induced *CXCL8* mRNA expression (Fig 11C and 11E) were both inhibited in cells treated with *SLC7A2* siRNA or with L-Lys.

## Discussion

**Fig 12:**
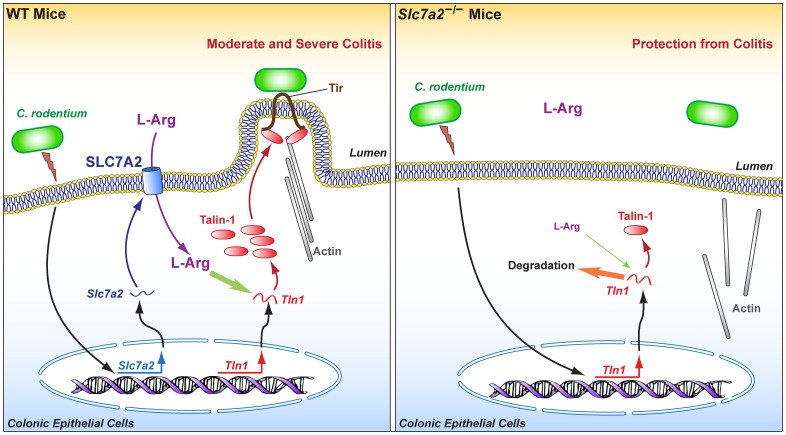
Model for the regulation of the intimate attachment of A/E pathogens to epithelial cells by SLC7A2 and the resulting effect on disease pathogenesis.

SLC7A2 is a master regulator of macrophage function notably by controlling the activity of the innate enzymes that use L-Arg as substrate, namely NOS2 and arginase [6, 25, 26]. Consequently, this transporter orchestrates the susceptibility of the host to parasites, including *Toxoplasma gondii* and *Schistosoma mansoni* [27], and to pathogenic bacteria such as *H*. *pylori* [9], and regulates adaptive immunity during infectious [9, 27] and inflammatory processes [10]. Herein, we now demonstrate that epithelial SLC7A2 plays a critical role in the attachment of intestinal pathogenic bacteria to colonocytes and to the formation of A/E lesions, thus triggering the initiation of the inflammatory cascade through chemokine production. This occurs through the effect of intracellular L-Arg that is required to increase mRNA stability of Talin-1, a focal adhesion protein involved in the intimate attachment of A/E pathogens (Fig 12). Using the murine intestinal pathogen *C*. *rodentium*, we showed that SLC7A2 is induced during the infection mainly in CECs and that colonization and inflammation were attenuated in *Slc7a2* ^–/–^mice, resulting in improved clinical and histological parameters. Our results showed that the difference in colonization between WT and *Slc7a2*-deficient animals was observed as soon as one-day post-infection and during the complete time course of the disease; this indicates that the improved colitis observed in *Slc7a2* ^–/–^mice is likely to be due to decreased colonization, since the level of bacterial burden is generally associated with the level of inflammation in this model [28]. Our data also highlight a previously unappreciated role for epithelial SLC7A2 in initiating the recruitment of myeloid cells in the infected mucosa and the development of Th1 and Th17 populations during the infectious process.

We evidenced herein that *Slc7a2* ^–/–^mice exhibit less colonic inflammation than WT animals after *C*. *rodentium* infection, whereas our previous work showed that *Slc7a2* ^–/–^mice have worsened colitis when treated with DSS when compared to WT mice [10]; this suggests that different mechanisms orchestrate A/E bacteria-induced mucosal inflammation and experimental colitis initiated by a chemical that destroys the surface of the epithelium. Similar differences in epithelial injury versus infectious models have been described. As examples, mice lacking the TNF-α receptor TNFRp55 or IFN-γ display an attenuation of DSS-induced colitis [29, 30], but when infected with *C*. *rodentium*, they exhibit enhanced colonic bacterial burden, worsened clinical and histological parameters, and more mucosal inflammation than WT mice [31, 32]. In the same way, the Th17 lineage is required for protection against *C*. *rodentium* [33], but *Il17a* ^−/−^ mice show reduced severity of colitis in the DSS model [34]. Moreover, we have described that SLC7A2 is induced in colonic macrophages after DSS treatment [10], but here we show that expression is in CECs, and is not significant in the lamina propria cells, during *C*. *rodentium* infection. Further, bone marrow transfers between WT and *Slc7a2* ^–/–^mice resulted in a *C*. *rodentium*-induced phenotype driven by the recipient genotypes of the animals, demonstrating that the hematopoietic pools do not play a major role in SLC7A2-mediated disease development. These data emphasize that SLC7A2 may play a different role in the gastrointestinal tract according to the type of cells expressing this protein and to the etiology of the inflammation, i.e. epithelial injury or infectious processes.

Although *in vitro* analysis has evidenced that epithelial restitution is supported by SLC7A2–dependent L-Arg uptake by increasing L-proline synthesis through the arginase 1 metabolic pathway [24], our data herein indicate that *Slc7a2*-deficient mice displayed less colitis than WT after *C*. *rodentium* infection. Because arginase 1 is induced during *C*. *rodentium* infection [35], our new findings suggest that the deleterious effect of SLC7A2 in this model exceeds its potential beneficial effect and is not dependent on arginase activity. We therefore reasoned that SLC7A2 may play a role in the adherence of *C*. *rodentium* to CECs. Indeed, decreased *C*. *rodentium* colonization and epithelial cell EspB content in *Slc7a2*-deficient mice, co-localization of SLC7A2 and *C*. *rodentium* in the infected mucosa, and enhanced intimate attachment of *C*. *rodentium* to cultured cells overexpressing SLC7A2 provide strong evidence to support this postulate. Moreover, our *in vivo* and *in vitro* studies (summarized in Fig 12) have demonstrated that SLC7A2 activity and L-Arg concentration are coupled with regulation of transcription and mRNA stability of *Tln1*, the gene encoding Talin-1 that is essential for the intimate attachment of A/E pathogens to CECs [16, 17].

In summary, we have shown that SLC7A2 facilitates the binding of *C*. *rodentium* to CECs *in vitro* and *in vivo*, thus initiating a strong innate immune/inflammatory response of enterocytes. Intriguingly, in addition to its role in metabolic L-Arg uptake by numerous cells [36, 37], constitutive SLC7A1 has been also shown to be the receptor for ectopic murine leukemia viruses [38]. However, in the present report, we demonstrate that SLC7A2 regulates bacterial attachment through a mechanism that depends on L-Arg, as we were able to recapitulate effects of *Slc7a2* interference by using L-Lys, a competitive inhibitor of L-Arg uptake. Because the difference in colon colonization with *C*. *rodentium* between WT and *Slc7a2* ^–/–^mice occurs as soon as day 1 post-infection, we propose that SLC7A2 plays a crucial role in the early stage of the disease, which is essential for *C*. *rodentium* pathogenesis. It has been reported that bacterial adhesion to CECs is associated with the activation of enteric innate Th17 response in the first few days of the disease [39]. Supporting the possibility that our data has a relevance to this immunological event, we showed that *Slc7a2*-deficient mice had decreased *Il-17* mRNA levels and less recruitment of CD4^+^, IL-17^+^ cells in the infected mucosa than in WT animals. Our investigations lead us to propose that *C*. *rodentium* favors its own pathogenesis by inducing the expression of SLC7A2 in epithelial cells, representing a striking example of the manipulation of cell signaling by a pathogenic bacterium.

## Materials and Methods

### Ethics statement

Experiments were conducted under protocol M/08/124 approved by the the IACUC of Vanderbilt University and the Research and Development Committee of the Veterans Affairs Tennessee Valley Healthcare System. Procedures were performed in accordance with institutional policies, AAALAC guidelines, the AVMA Guidelines on Euthanasia (CO_2_ asphyxiation followed by cervical dislocation), NIH regulations (Guide for the Care and Use of Laboratory Animals), and the United States Animal Welfare Act (1966).

### Bacteria

EPEC strain E2348/69 was maintained on Luria-Bertani (LB) agar plates. *C*. *rodentium* DBS100 [40] and the isogenic mutants *escN*, *espF*, and *espG* [19, 41, 42] were maintained on McConkey agar plates and cultured overnight at 37°C in LB broth; this culture was then diluted to OD_600_ = 0.1 in LB broth or in cell culture medium to the exponential growth phase. These bacteria were used to infect mice or cells [35, 43]. The bacterial concentration was estimated to be 5 X 10^8^ bacteria/ml per OD unit at 600 nm, as calculated by plating.

### Animals and *C*. *rodentium* colitis

C57BL/6 and C57BL/6 *Slc7a2* ^–/–^mice were house-bred as described [9, 10]. Age-matched male WT and mutant mice (7–11 weeks) were infected by oral gavage with 0.1 ml of LB broth containing 5 X 10^8^ bacteria. Animals were monitored daily and mice that showed extreme distress, became moribund, or lost more than 20% of initial body weight were euthanized. After sacrifice, colons were removed, measured, cut longitudinally, cleaned, weighed, and Swiss-rolled for histology. Three proximal and distal 2 mm pieces were used for RNA and protein extraction and to determine colonization by plating serial dilution of ground tissues.

### Bone marrow transplantation

Bone marrow was harvested from the femurs and tibias of WT and *Slc7a2* ^–/–^mice as described [44]. Hematopoietic cells (10^6^) were injected retro-orbitally into 5-week-old WT and *Slc7a2* ^−/−^mice after each were irradiated with a single dose of 900 rads. The mice were fed autoclaved food and water, with the latter supplemented with 1.1 g/L neomycin sulfate and 125 mg/L polymyxin B for one-week pre-transplant and two-weeks post-transplant. Mice were infected with *C*. *rodentium* eight weeks after transplantation.

For genotyping, DNA extraction from a sample tissue of spleen and PCR were performed using EZ Fast PCR Genotyping Kit (EZ Bioresearch) and the primers for *Slc7a2* and *neo* genes (S2 Table).

### Isolation of colonic cells

CECs were isolated by a dissociation and dispersion method [24, 43, 45]. Briefly, colons were removed, cut open longitudinally, cleaned, cut into 2–3 mm pieces, and incubated in DTT (3 mM) and EDTA (0.5 mM). After one hour, samples were vigorously shaken in PBS and filtered through 70-μm nylon mesh. The purity of the epithelial cells was assessed by flow cytometry as described [24].

Cells from the lamina propria were isolated by enzymatic digestion using a dispase and collagenase method as reported [10, 44]. Briefly, mouse colons were removed, opened longitudinally, cleaned, cut into 1 mm pieces, and incubated in dispase (1 mg/ml) and collagenase A (0.25 mg/ml) for 20 min. Cells were filtered through a 70-μm strainer, centrifuged, and counted. Cells were then labeled with a biotin-conjugated anti-mouse F4/80 antibody (1:200; CALTAG Laboratories), washed, and incubated with streptavidin conjugated with magnetic beads (BD Biosciences). Cell suspensions were applied to an IMagnet (BD Biosciences) for 6 min at room temperature; F4/80^−^ fractions were carefully removed and this selection procedure was repeated 3 times, before collecting the F4/80^+^ fraction.

### Immunophenotyping

Isolated cells from the lamina propria were analyzed by flow cytometry using labelled Abs (S3 Table). For intracellular proteins the cell suspensions were incubated in complete RPMI 1640 medium containing GolgiPlug (BD Biosciences) for 4 h before staining [10, 46]. Single cell suspensions from mesenteric lymph nodes were cultured in complete RPMI 1640 medium with 5 μg/ml anti-CD3. After 24 h, 1 μg/ml soluble anti-CD28 was added for 2 more days; cells were then stimulated with 20 ng/ml phorbol 12-myristate 13-acetate and 1 μg/ml ionomycin in the presence of GolgiPlug for 4 h [10, 46].

### Histologic score

Swiss-rolled colons were formalin-fixed and paraffin-embedded, and 5 μm sections were stained with H&E and examined in a blinded manner by a gastrointestinal pathologist (M.B.P.). The histologic injury score (0–21) was the sum of acute and chronic inflammation (0–3 for each) scores multiplied by extent of inflammation (0–3) plus the epithelial injury score (0–3), as described [43]. Histological sections were also stained with Periodic acid–Schiff that is specific for goblet cells [47].

### Cells, transduction, transfection, and infection

YAMC cells were maintained under permissive growth conditions in DMEM medium supplemented with 10% fetal bovine serum, 2 mM glutamine, 50 μg/ml gentamicin, 100 U/ml penicillin, 100 μg/ml streptomycin, and 5 U/ml IFN-γ in a humidified incubator with 5% CO_2_ at 33°C [24, 43]. These cells were plated in 96-well plates at 33°C for 20 h and hexadimethrine bromide (8 μg/ml) was added to the cells to enhance transduction efficiency. Lentiviral particles containing *Slc7a2* or control shRNAs (Sigma) were added at an MOI of 5 and incubated for an additional 20 h at 33°C. Transduced cells were selected in complete medium with 10 μg/ml puromycin, which was replaced every 3–4 days for 3 weeks [24]. YAMC cells, transduced or not, were then transferred at 37°C in IFN-γ-free medium for 24 h before infection with *C*. *rodentium*.

Caco-2 and HEK 293T cells were grown in DMEM containing 10% fetal bovine serum, 2 mM glutamine, 100 U/ml penicillin, and 100 μg/ml streptomycin. These cells were plated at 5 X 10^5^ cells per well in 6-well plates at 37°C for 20 h. Medium was changed to Opti-MEM I Reduced-Serum Media (Invitrogen) and cells were transfected using Lipofectamine 2000 (Life Technologies) with *i*) 100 nM ON-TARGETplus siRNAs (Dharmacon) directed against human *SLC7A2* or scrambled siRNAs, or *ii*) 1 μg of the pLX304 plasmid vector expressing or not the human *SLC7A2* gene. After 6 h, cells were washed, maintained 24–48 h in fresh medium, and then infected with *C*. *rodentium* or EPEC, in the presence or absence of 40 mM L-Lys.

### mRNA analysis

RNA from colonic tissues and cells was extracted using the RNeasy Mini Kit (Qiagen) as described [10]. RNA from isolated CECs, F4/80^+^, and F4/80^−^ cells was purified using the 5 PRIME PerfectPure RNA 96 Cell CS Kit (5 PRIME). Total RNA (1 μg) was reverse transcribed using an iScript cDNA synthesis kit (Bio-Rad) and oligo(dT) primers. Each PCR was performed with 2 μl of cDNA, iQ^™^ SYBR Green Supermix (Bio-Rad), and primers described in S2 Table.

RNA *in situ* hybridization was performed using the 2.0 HD Brown FFPE Reagent Kit (Advanced Cell Diagnostics). Briefly, 5 μm sections from formalin-fixed paraffin embedded Swiss-rolled tissues were heated for 1 h at 60°C, incubated twice in xylene for 5 min, washed twice in 100% ethanol for 1 min, dried for 5 min at room temperature, treated with H_2_O_2_ for 10 min, boiled in target retrieval reagent, and incubated with protease solution for 30 min at 40°C. Sections were then incubated with a custom-made *Slc7a2* probe overnight, washed, and signals were amplified using a six-step protocol according to the manufacturer’s instructions. Color was developed using 3, 3'-diaminobenzidine substrate and slides were counterstained with hematoxylin.

To study *Tln1* mRNA stability, YAMC cells were first maintained in L-Arg-free DMEM (GIBCO Life Technologies) supplemented with 0.1 mM L-Arg for 4 h, washed once, and switched to fresh L-Arg-free DMEM containing or not 0.1 mM L-Arg. Actinomycin D (10 μg/ml) was added directly to the cells cultured with or without L-Arg. After different times, *Tln1* mRNA was analyzed by RT-real time PCR.

### Immunostaining and immunofluorescence

Sections (5 μm) from formalin-fixed paraffin-embedded Swiss-rolled tissues were deparaffinized, treated with citrate buffer for antigen retrieval, and blocked in 5% goat serum for 1 h at room temperature. For immunohistochemistry, slides were subsequently incubated at 4°C for 16 h with mouse monoclonal anti-EspB (1:100; tgcBIOMICS) and MACH2 Mouse HRP polymer was used (Biocare Medical). Color was developed using 3, 3'-diaminobenzidine substrate and slides were counterstained with hematoxylin. Slides were washed, dried, mounted using mounting medium, and visualized using a Nikon E800 fluorescence microscope and Spot RT Slider digital camera (Diagnostic Instruments); images were analyzed using Spot Advanced software (Diagnostic Instruments).

For immunofluorescence, slides were blocked with Background Sniper (Biocare Medical) and subsequently incubated with *i*) rabbit polyclonal anti-SLC7A2 Ab (Sigma; 1:200), Alexa Fluor 555-labeled goat anti-rabbit IgG (1:400; Life Technologies), Fab Fragment Donkey anti-rabbit IgG (Jackson ImmunoResearch) in 5% goat serum, rabbit polyclonal anti-*C*. *koseri*, which has been shown to cross-react with *C*. *rodentium* (Abcam; 1:50), and Alexa Fluor 488-labeled goat anti-rabbit IgG (1:400; Life Technologies), or *ii*) mouse monoclonal anti-EspB (1:200; tgcBIOMICS), Alexa Fluor 555-labeled goat anti-murine IgG (1:500), rabbit monoclonal anti-Talin-1 Ab (Cell Signaling; 1:200), and Alexa Fluor 488-labeled goat anti-rabbit IgG (1:700). Slides were washed, dried, mounted using DAPI (for nuclear staining) containing mounting medium, and then visualized by confocal microscopy (FV1000; Olympus).

### FAS Test

YAMC cells and Caco-2 cells were plated on coverslips and infected with *C*. *rodentium* or EPEC at an MOI 10 for 6 h, respectively. Cells were washed with PBS, fixed with 2.5% glutaraldehyde, permeabilized in 0.1% triton X, and stained with Phalloidin CF543 (1:50; Biotium) for 1 h. Coverslips were washed with PBS, mounted on glass slides, and visualized by confocal microscopy.

### Measurement of cytokine concentrations

Colonic tissues were lysed in Cell Lytic Mammalian Tissue Lysis Extraction Reagent (Sigma) and analyzed using the 25-analyte MILLIPLEX MAP Mouse Cytokine/Chemokine Magnetic Bead Panel (EMD Millipore) on a FLEXMAP 3D instrument (Luminex) [9, 10]. Data were standardized to tissue protein concentrations measured by the BCA Protein Assay Kit (Pierce).

### Western blotting

Colonic tissues or cells were lysed in Cell Lytic Mammalian Tissue Lysis Extraction Reagent (Sigma) containing the EDTA-free Protease Inhibitor Cocktail Set III, and the Phosphatase Inhibitor Set I (Millipore), and disrupted by sonication at 40 W. Protein concentrations were determined by the BCA Protein Assay Kit (Pierce). Total proteins (80 μg per lane) were separated on 10% polyacrylamide gels (Bio-Rad) and transferred onto PVDF membranes. Monoclonal anti-EspB (1:1000), rabbit monoclonal anti-Talin-1 Ab (1:2000), rabbit polyclonal anti-ACTN1 Ab (1:4000; Cell Signaling), rabbit polyclonal anti-SLC7A2 Ab (1:2000; Abcam), and mouse monoclonal anti-β-actin (1:10,000; Sigma) were used as described [9, 10].

### L-arginine uptake

Freshly collected colonic tissues from uninfected and *C*. *rodentium*-infected mice were maintained in DMEM containing 5 μM [^14^C]-L-Arg (specific activity, 346 mCi/mmol) for 5 min. Tissues were then washed three times with PBS, lysed with RIPA buffer, and supernatants were collected. Tissue lysates were mixed with scintillation fluid and the [^14^C] content was determined in a scintillation counter. Protein was measured in tissue lysates by the bicinchoninic acid method as described previously [10]. L-Arg transport values were expressed as pmol [^14^C]-L-Arg/min/mg protein.

### L-arginine measurement

Blood was collected from WT and *Slc7a2* ^–/–^mice at the time of euthanasia via cardiac puncture and serum was separated as described [8, 10]. Serum were diluted 1:5 with acetonitrile and derivatized by sequential addition of 50 mM sodium carbonate and 2% benzoyl chloride (v/v in acetonitrile). An internal standard solution (13C6-benzoyl chloride derivatized arginine at a concentration of 8.7 pg/μl in 20% acetonitrile in water with 2% sulfuric acid) was also added to the samples. LC–MS analysis was performed using a Waters Acquity UPLC (Milford, MA, USA) coupled to a SCIEX 6500+ QTrap mass spectrometer (Framingham, MA, USA) operating in multiple reaction monitoring mode [48].

### Statistical analysis

Quantitative data are shown as the mean ± SEM. Statistical analyses were performed with Prism version 5.0c (GraphPad Software). Student’s *t* test was performed for comparison between two groups. Analysis of variance with the Student-Newman-Keuls post-hoc multiple comparisons test was performed to compare multiple groups. Survival was analyzed by the Log-rank (Mantel-Cox) Test.

## Supporting Information

S1 Fig Expression and activity of SLC7A1 and SLC7A2. (**A**) Swiss-rolled colons from *Slc7a2* ^–/–^animals that were uninfected (Ctrl) or infected with *C*. *rodentium* (*C*. *rod*), were probed for *Slc7a2* RNA, and visualized using DAB. Scale bar, 50 μm. Representative images of 3 mice. (**B**) *Slc7a1* mRNA levels were analyzed in colon tissues by real-time PCR (*n* = 3 for Ctrl and *n* = 8 for *C*. *rodentium*-infected; ns = not significant). (**C**) Western blot for SLC7A2 in isolated CECs. (**D**) *Ex vivo* measurement of L-Arg uptake by colonic tissues and L-Arg concentration in serum. **P < 0.01, ***P < 0.001.
(TIF)

S2 Fig Expression of *Slc7a2* in cultured murine CECs. YAMC cells were infected or not with *C*. *rodentium* (*C*. *rod*) or with the isogenic mutants for 4 h. The expression of *Slc7a2* (**A**) as well as *Cxcl1* and *Cxcl2* (**B**) mRNA was analyzed. *P < 0.05 compared to Ctrl, §*P* < 0.05 compared to *C*. *rodentium*-infected cells; *n* = 3 independent experiments.
(TIF)

S3 Fig Goblet cells in *C*. *rodentium* infection. (**A**) Colons from WT and *Slc7a2* ^–/–^mice, that were uninfected (Ctrl) or infected with *C*. *rodentium* (*C*. *rod*), were stained for goblet cells. Representative images are shown. Scale bar, 200 μm (**B**) Percent of mucosa with loss of goblet cells; **P < 0.01. For (*A*) and (*B*), *n* = 5–7 mice for Ctrl and *n* = 5–9 for *C*. *rodentium*-infected mice.
(TIF)

S4 Fig Genetic assessment of transplantation. After bone marrow transplantation, DNA from spleen of recipient animals was analyzed by PCR for the genes *Slc7a2* and *neo*. Representative PCR gel of two animals in each condition. The predominant band is that of donor mice.
(TIF)

S5 Fig Expression of *Slc7a2* in transduced and transfected cells. mRNA levels of *Slc7a2* in HEK 293 cells expressing pLX304 or p*Slc7a2* (**A**), and in YAMC cells transduced with Ctrl or *Slc7a2* shRNA and then infected with *C*. *rodentium* (*C*. *rod*) (**B**). **P < 0.01, ***P < 0.001; *n* = 3–5 independent experiments. (**C**) Levels of SLC7A2 by Western blotting.
(TIF)

S1 Table Cytokine and chemokine concentrations in colonic tissues. (DOCX)

S2 Table Sequences of primers for PCR. (DOCX)

S3 Table Abs used for flow cytometry. (DOCX)
