# Supplementary figures and images for: The L-Arginine Transporter Solute Carrier Family 7 Member 2 Mediates the Immunopathogenesis of Attaching and Effacing Bacteria
Source: PLoS Pathog. 2016 Oct 26;12(10):e1005984. doi: 10.1371/journal.ppat.1005984 (PMC5081186; doi:10.1371/journal.ppat.1005984)

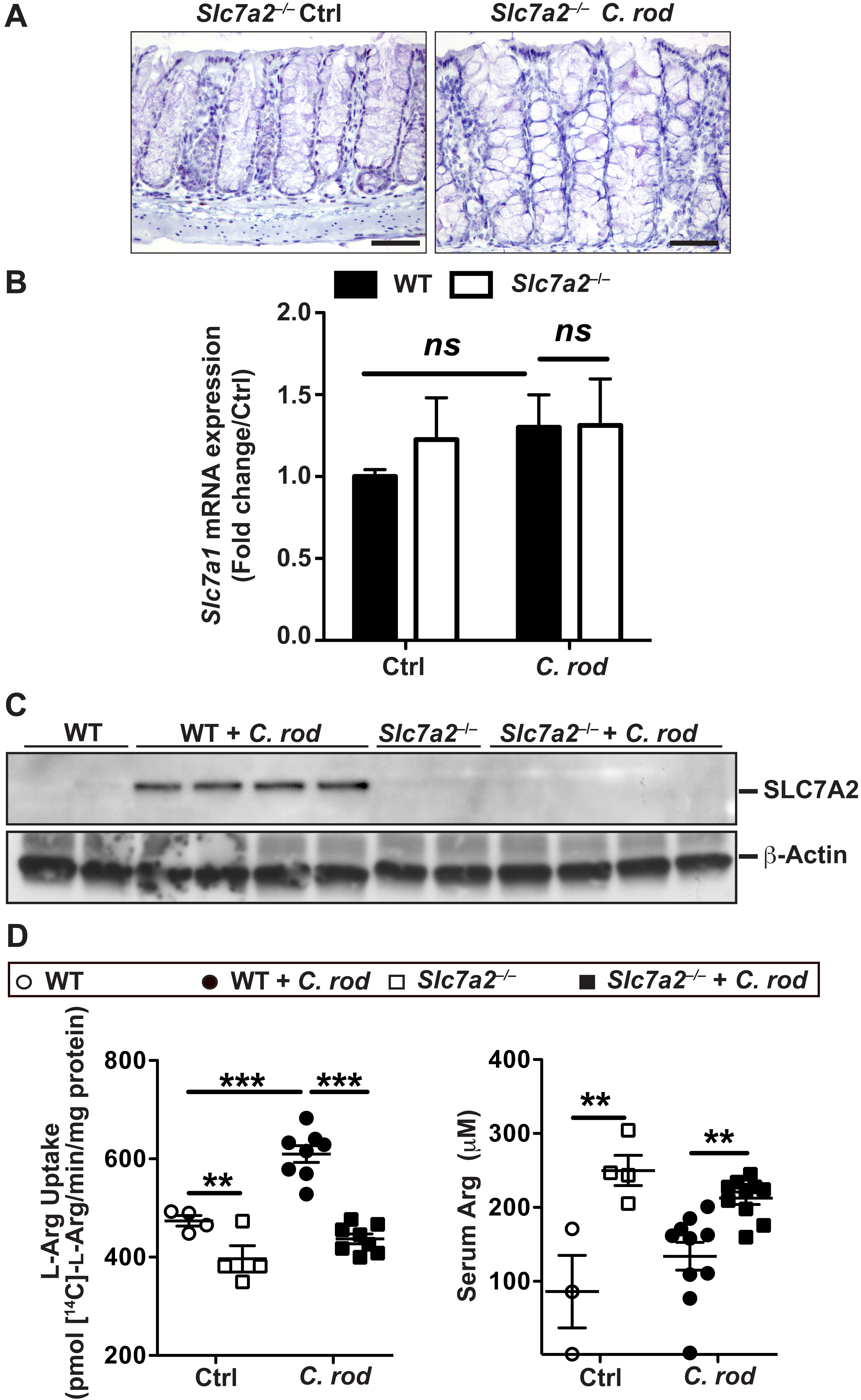

Supplement: S1 Fig — (A) Swiss-rolled colons from Slc7a2 –/–animals that were uninfected (Ctrl) or infected with C. rodentium (C. rod), were probed for Slc7a2 RNA, and visualized using DAB. Scale bar, 50 μm. Representative images of 3 mice. (B) Slc7a1 mRNA levels were analyzed in colon tissues by real-time PCR (n = 3 for Ctrl and n = 8 for C. rodentium-infected; ns = not significant). (C) Western blot for SLC7A2 in isolated CECs. (D) Ex vivo measurement of L-Arg uptake by colonic tissues and L-Arg concentration in serum. **P < 0.01, ***P < 0.001. (TIF) [file ppat.1005984.s001.tif]

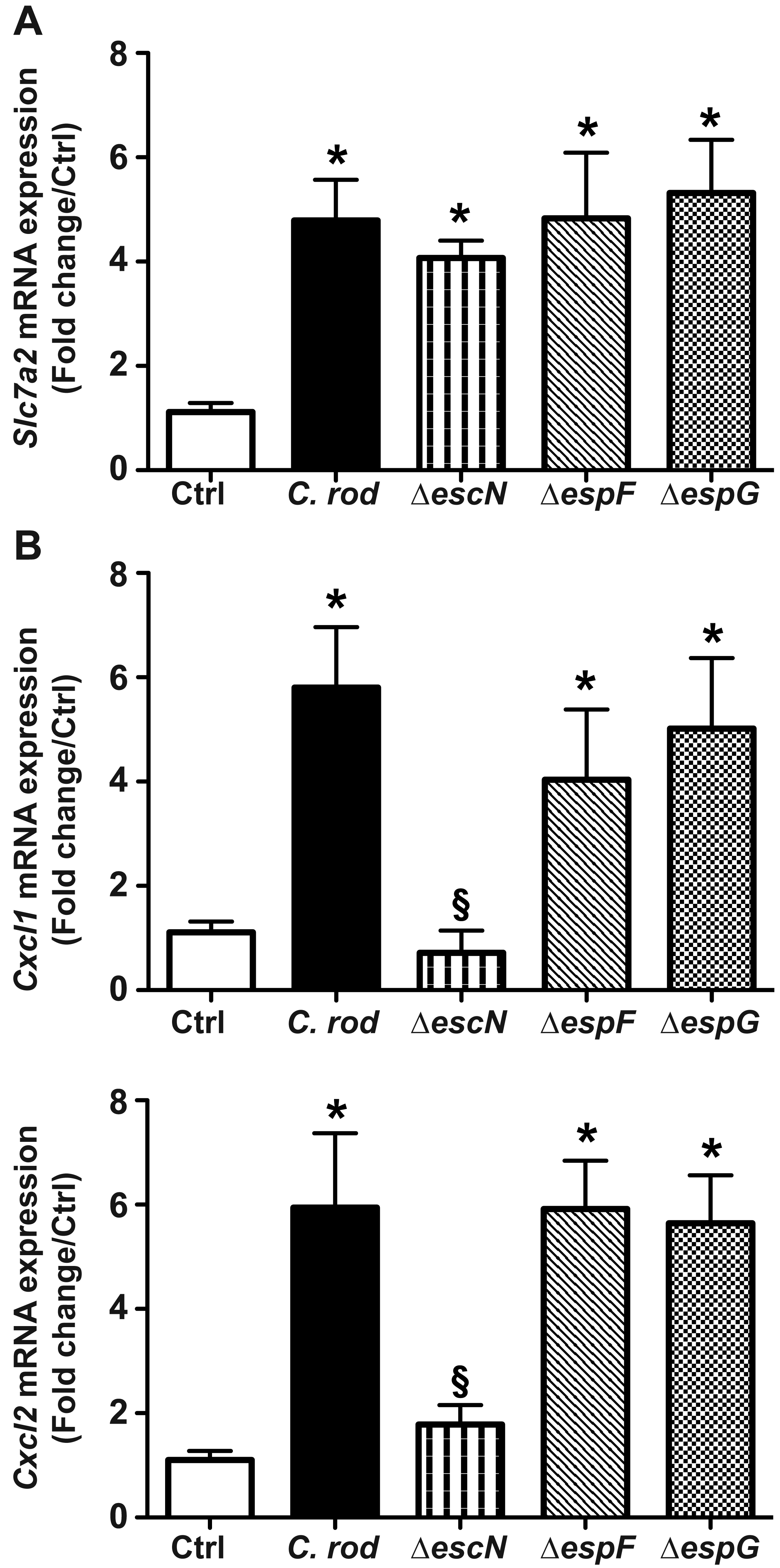

Supplement: S2 Fig — YAMC cells were infected or not with C. rodentium (C. rod) or with the isogenic mutants for 4 h. The expression of Slc7a2 (A) as well as Cxcl1 and Cxcl2 (B) mRNA was analyzed. *P < 0.05 compared to Ctrl, §P < 0.05 compared to C. rodentium-infected cells; n = 3 independent experiments. (TIF) [file ppat.1005984.s002.tif]

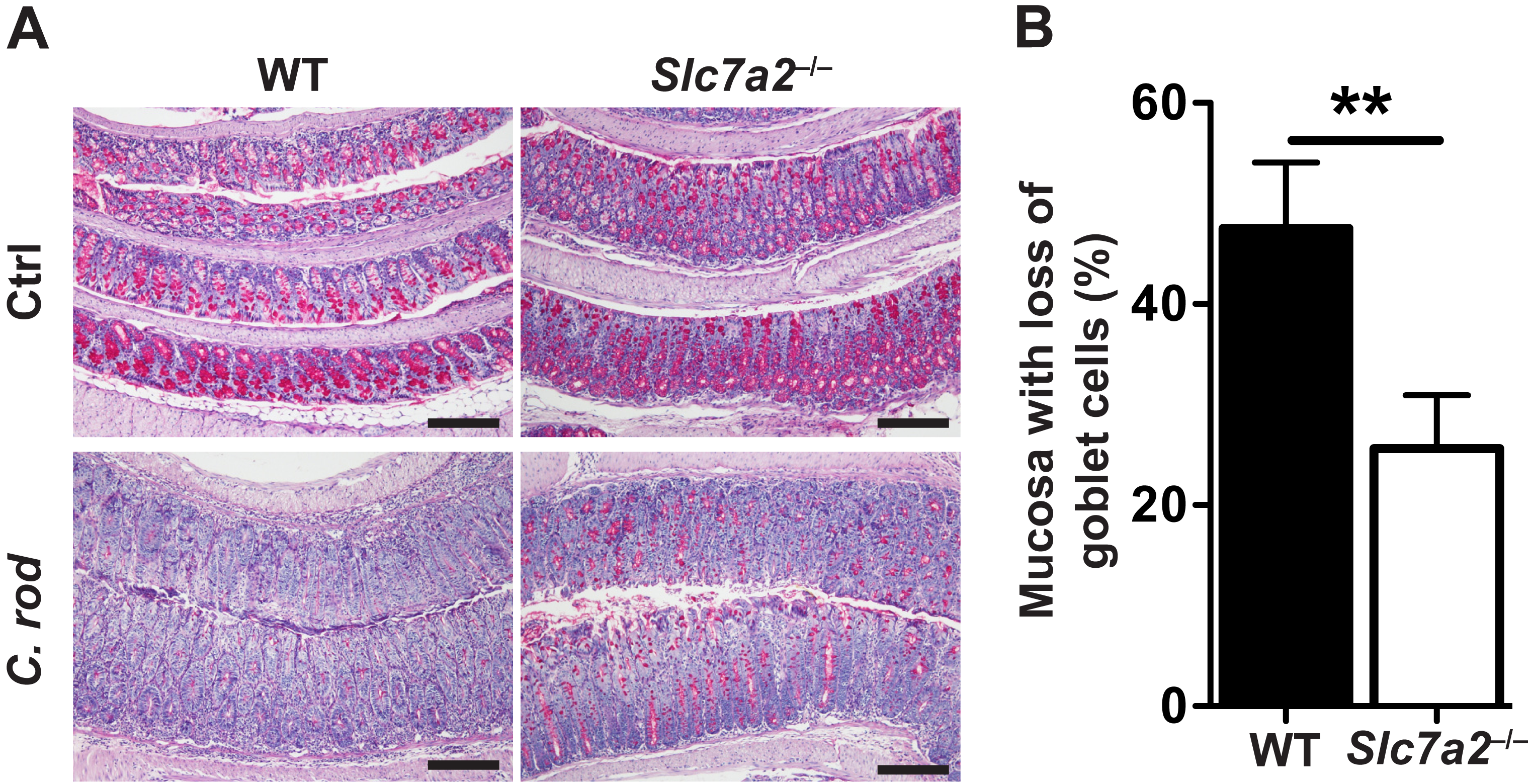

Supplement: S3 Fig — (A) Colons from WT and Slc7a2 –/–mice, that were uninfected (Ctrl) or infected with C. rodentium (C. rod), were stained for goblet cells. Representative images are shown. Scale bar, 200 μm (B) Percent of mucosa with loss of goblet cells; **P < 0.01. For (A) and (B), n = 5–7 mice for Ctrl and n = 5–9 for C. rodentium-infected mice. (TIF) [file ppat.1005984.s003.tif]

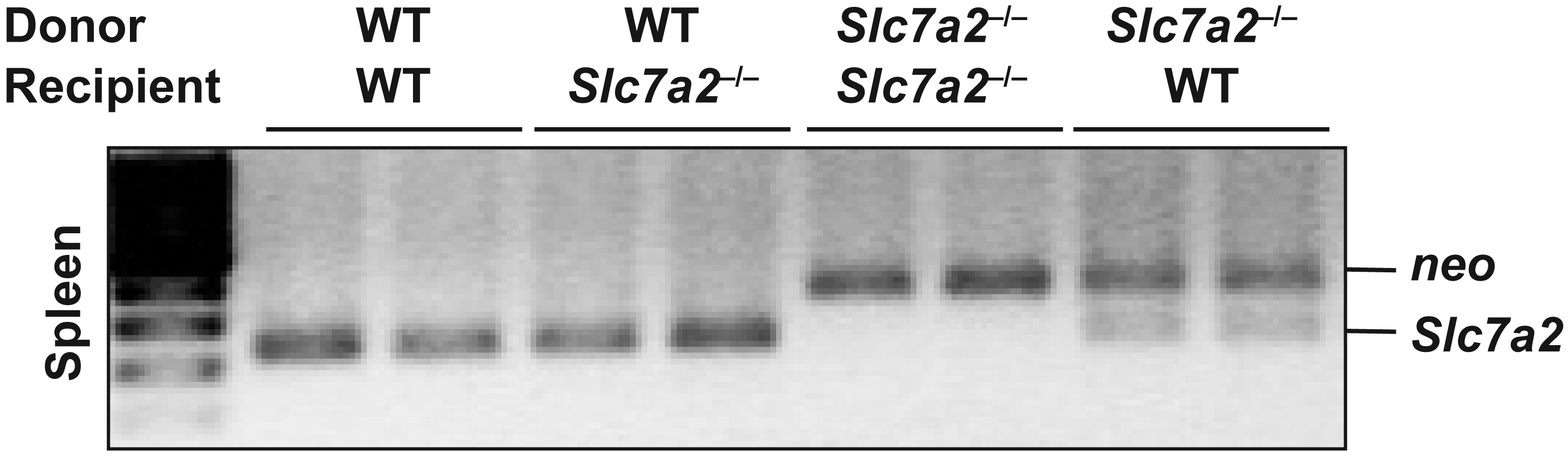

Supplement: S4 Fig — After bone marrow transplantation, DNA from spleen of recipient animals was analyzed by PCR for the genes Slc7a2 and neo. Representative PCR gel of two animals in each condition. The predominant band is that of donor mice. (TIF) [file ppat.1005984.s004.tif]

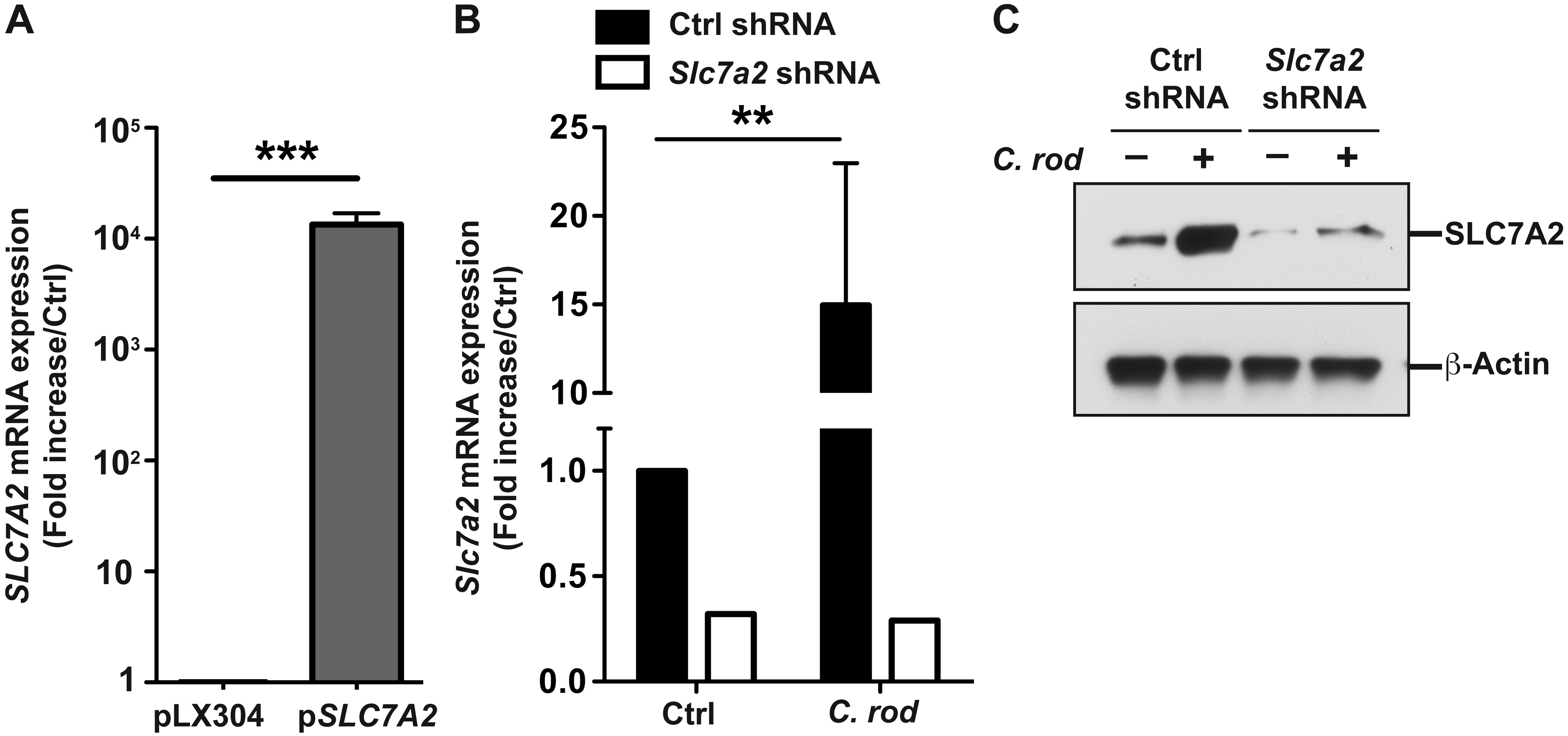

Supplement: S5 Fig — mRNA levels of Slc7a2 in HEK 293 cells expressing pLX304 or pSlc7a2 (A), and in YAMC cells transduced with Ctrl or Slc7a2 shRNA and then infected with C. rodentium (C. rod) (B). **P < 0.01, ***P < 0.001; n = 3–5 independent experiments. (C) Levels of SLC7A2 by Western blotting. (TIF) [file ppat.1005984.s005.tif]
