# Supplementary material for: The L-Arginine Transporter Solute Carrier Family 7 Member 2 Mediates the Immunopathogenesis of Attaching and Effacing Bacteria
Source: PLoS Pathog. 2016 Oct 26;12(10):e1005984. doi: 10.1371/journal.ppat.1005984 (PMC5081186; doi:10.1371/journal.ppat.1005984)
Supplement: S1 Table — (DOCX) [file ppat.1005984.s006.docx]

**S1 Table. Cytokine and chemokine concentrations in colonic tissues**

| Analyte | WT Ctrl | *Slc7a2*^–/–^ Ctrl | WT *C. rod* | *Slc7a2*^–/–^ *C. rod* |
| --- | --- | --- | --- | --- |
| CCL2 | 4.3 ± 1.6 | 13.2 ± 3.9 | 231.4 ± 27.8*** | 63.6 ± 14.1*††† |
| CCL3 | 2.2 ± 0.3 | 5.4 ± 1.4 | 32.7 ± 4.8*** | 12.5 ± 3.2††† |
| CCL4 | 7.6 ± 3.2 | 24.7 ± 7.0* | 74.2 ± 8.7*** | 31.4 ± 6.7*††† |
| CCL5 | 28.8 ± 8.6 | 54.6 ± 15.9 | 53.5 ± 4.8 | 34.3 ± 5.3 |
| CXCL1 | 4.5 ± 0.6 | 11.4 ± 1.9 | 156.0 ± 22.6*** | 85.9 ± 22.5**†† |
| CXCL2 | 5.0 ± 0.6 | 7.1 ± 1.2 | 530.2 ± 88.9*** | 130.2 ± 61.6††† |
| CXCL10 | 34.8 ± 7.9 | 61.6 ± 9.5 | 524.5 ± 18.6*** | 201.3 ± 39.4*††† |
| G-CSF | 0.8 ± 0.1 | 2.9 ± 1.6 | 95.7 ± 24.4*** | 12.0 ± 2.8††† |
| GM-CSF | 0.9 ± 0.1 | 1.0 ± 0.9 | 3.9 ± 0.8 | 2.9 ± 0.7 |
| IL-1α | 4.8 ± 0.8 | 5.1 ± 0.9 | 17.3 ± 4.2* | 3.7 ± 0.4†† |
| IL-1ß | 3.9 ± 2.2 | 5.4 ± 1.1 | 28.7 ± 4.4*** | 10.3 ± 2.1††† |
| IL-2 | 0.8 ± 0.1 | 0.8 ± 0.1 | 0.7 ± 0.1 | 0.6 ± 0.1 |
| IL-4 | 0.1 ± 0.1 | 0.1 ± 0.1 | 0.1 ± 0.0 | 0.1 ± 0.1 |
| IL-5 | 0.2 ± 0.1 | 0.8 ± 0.4 | 0.2 ± 0.1 | 0.3 ± 0.1 |
| IL-6 | 0.1 ± 0.0 | 0.1 ± 0.1 | 99.7 ± 38.5** | 10.7 ± 3.2††† |
| IL-7 | 2.6 ± 0.3 | 4.3 ± 0.5 | 4.2 ± 0.9 | 2.7 ± 0.3 |
| IL-9 | 9.6 ± 2.4 | 13.2 ± 1.6 | 19.5 ± 3.6 | 10.2 ± 1.7 |
| IL-10 | 1.1 ± 0.2 | 1.7 ± 0.4 | 1.2 ± 0.2 | 0.9 ± 0.2 |
| IL-12p40 | 0.7 ± 0.3 | 1.7 ± 0.3 | 1.6 ± 0.3 | 0.6 ± 0.1 |
| IL-12p70 | 1.0 ± 0.3 | 1.1 ± 0.3 | 1.7 ± 0.4 | 0.4 ± 0.1† |
| IL-13 | 3.2 ± 0.4 | 3.9 ± 1.0 | 3.1 ± 0.6 | 2.6 ± 0.5* |
| IL-15 | 6.9 ± 1.4 | 10.8 ± 2.4 | 5.8 ± 1.4 | 4.2 ± 0.6 |
| IL-17 | 1.2 ± 0.1 | 1.4 ± 0.2 | 8.3 ± 0.6*** | 4.7 ± 1.2††† |
| TNF-α | 0.5 ± 0.1 | 0.8 ± 0.2 | 20.5 ± 1.1*** | 5.9 ± 1.5††† |
| IFN-γ | 1.8 ± 0.2 | 1.6 ± 0.2 | 6.0 ± 1.0** | 1.9 ± 0.3††† |

Values represent the mean ± SEM of the cytokine/chemokine concentrations (pg/mg total protein) in the colonic tissues, measured by Luminex assay. Ctrl, uninfected mice; *C. rod*, *C. rodentium*-infected mice. **P* < 0.05, ***P* < 0.01, ****P* < 0.001 vs*.* WT ctrl; †*P* < 0.05, ††*P* < 0.01, †††*P* < 0.001 vs*.* WT mice infected with *C. rodentium*; *n* = 5 uninfected and *n* = 12-14 infected mice for each genotype.
