# Supplementary material for: The L-Arginine Transporter Solute Carrier Family 7 Member 2 Mediates the Immunopathogenesis of Attaching and Effacing Bacteria
Source: PLoS Pathog. 2016 Oct 26;12(10):e1005984. doi: 10.1371/journal.ppat.1005984 (PMC5081186; doi:10.1371/journal.ppat.1005984)
Supplement: S2 Table — (DOCX) [file ppat.1005984.s007.docx]

**S2 Table. Sequences of primers for PCR**

| Species | Target genes | Sequence (5’–3’) | References |
| --- | --- | --- | --- |
| Mouse | *Ifn-γ* | F: GGCCATCAGCAACAACATAAGCGT | [1] |
|  |  | R: TGGGTTGTTGACCTCAAACTTGGC |  |
| Mouse | *Tnf-α* | F: CTGTGAAGGGAATGGGTGTT | [1] |
|  |  | R: GGTCACTGTCCCAGCATCTT |  |
| Mouse | *Il-17A* | F: ATCCCTCAAAGCTCAGCGTGTC | [1] |
|  |  | R: GGGTCTTCATTGCGGTGGAGAG |  |
| Mouse | *G-csf* | F: gctgtgcctcgaccccatgg | This work |
|  |  | R: Agggccaatgtccagggcct |  |
| Mouse | *Ccl2* | F: TACTCATTAACCAGCAAGAT | This work |
|  |  | R: TTGAGGTGGTGGTGGAA |  |
| Mouse | *Ccl3* | F: CTCCCAGCCAGGTGTCATT | This work |
|  |  | R: GGCATTCAGTTCCAGGTCAG |  |
| Mouse | *Ccl4* | F: ACATACTCATTGACCCAGGGCTC | This work |
|  |  | R: TAGGCTCTGACCCTCCCACTTC |  |
| Mouse | *Ccl5* | F: CATATGGCTCGGACACCA | This work |
|  |  | R: ACACACTTGGCGGTTCCT |  |
| Mouse | *Cxcl1* | F: GCTGGGATTCACCTCAAGAA | [1] |
|  |  | R: CTTGGGGACACCTTTTAGCA |  |
| Mouse | *Cxcl2* | F: gccaAGGGTTGACTTCA | [1] |
|  |  | R: TGTCTGGGCGCAGTG |  |
| Mouse | *Slc7a2* | F: TGATCCGGAGAAAAATTGTCA | [1] |
| Mouse | *Slc7a1* | R: CCCAAATTCAGCATAGCAAA  F: AAAGCAGCCCTTCTCC  R: AGGCTCACTAGCCATCTGGA | [1] |
| Mouse | *Tln1* | F: CCAAAAGCGACCGGGAGAAG  R: TCGTACACCATGGTAGATGGC | This work |
| Rat/Mouse | *ß-actin* | F: CCAGAGCAAGAGAGGTATCC | [1] |
| Human  Human | *SLC7A2*  *CXCL8* | R: CTGTGGTGGTGAAGCTGTAG  F: GTTGACTGCAGGGGTCATTT  R: ACATTTGGGCTGGTCGTAAG  F: TCTTGGCAGCCTTCCTGATT  R: TCTTTAGCACTCCTTGGCAAAAC | [2]  This work |
| – | *neo* | F: TGATTGAACAAGATGGATTGC  R: ATCAGCCATGATGGATACTTTCT | This work |

1. Singh K, Coburn LA, Barry DP, Asim M, Scull BP, Allaman MM, et al. Deletion of cationic amino acid transporter 2 exacerbates dextran sulfate sodium colitis and leads to an IL-17-predominant T cell response. Am J Physiol Gastrointest Liver Physiol. 2013; 305(3):G225-40. Epub 2013/05/25. doi: 10.1152/ajpgi.00091.2013. PubMed PMID: 23703655; PubMed Central PMCID: PMC3742860.

2. Chaturvedi R, Asim M, Hoge S, Lewis ND, Singh K, Barry DP, et al. Polyamines impair immunity to *Helicobacter pylori* by inhibiting L-arginine uptake required for nitric oxide production. Gastroenterology. 2010; 139(5):1686-98. Epub 2010/07/06. doi: 10.1053/j.gastro.2010.06.060. PubMed PMID: 20600019; PubMed Central PMCID: PMC2967614.
