# Supplementary material for: The L-Arginine Transporter Solute Carrier Family 7 Member 2 Mediates the Immunopathogenesis of Attaching and Effacing Bacteria
Source: PLoS Pathog. 2016 Oct 26;12(10):e1005984. doi: 10.1371/journal.ppat.1005984 (PMC5081186; doi:10.1371/journal.ppat.1005984)
Supplement: S3 Table — (DOCX) [file ppat.1005984.s008.docx]

**S3 Table. Abs used for flow cytometry**

| Target | Marker | Fluorochrome | Isotype/Catalog Number |
| --- | --- | --- | --- |
| Granulocytes | Gr1 | Phycoerythrin (PE) | Rat IgG2b (108407)* |
| Macrophages | F4/80 | Allophycocyanin (APC) | Rat IgG2a (123116)* |
| Dendritic cells | CD11c | Allophycocyanin-Cy7 (APC-Cy7) | Armenian Hamster IgG (117324)* |
| T helper cells | CD4 | Peridinin-chlorophyll protein (PerCp)-Cy5.5 | DA/HA IgG2a (550954)† |
| T helper 1 | IFN-γ | Fluorescein (FITC) | Rat IgG1 (554411)† |
| T helper 17 | IL-17 | PE | Mouse IgG1 (560486)† |

*Abs were from BioLegend.

†Abs were from BD Biosciences.
